# Discovery of VU6081679, a Novel Positive Allosteric Modulator of Metabotropic Glutamate Receptor 8 with Efficacy in a Preclinical Model of Parkinson’s Disease

**DOI:** 10.1021/acschemneuro.6c00364

**Published:** 2026-07-16

**Authors:** Alexa E. Richardson, Anthony S. Ferranti, Li Peng, Xia Lei, Allie Han, Aidong Qi, Alice L. Rodriguez, Sichen Chang, Snehal Sant, Irene Zagol-Ikapitte, Srinivasan Krishnan, Analisa Thompson Gray, Joshua C. Wilkinson, Sri H. Kethanapalli, Hyekyung P. Cho, Kayla J. Temple, Dehui Mi, Emily L. Days, Joshua A. Bauer, N. Kithmini Wijesiri, Benjamin P. Brown, Olivier Boutaud, Carrie K. Jones, Darren W. Engers, Craig W. Lindsley, Colleen M. Niswender, Katherine E. Crocker

**Affiliations:** † Warren Center for Neuroscience Drug Discovery, 5718 Vanderbilt University, Nashville, Tennessee 37232, United States; ‡ Department of Pharmacology, 12327 Vanderbilt University School of Medicine, Nashville, Tennessee 37232, United States; § Vanderbilt Institute for Therapeutic Advances, Vanderbilt University School of Medicine, Nashville, Tennessee 37232, United States; ∥ Vanderbilt Institute of Chemical Biology, Vanderbilt University, Nashville, Tennessee 37232, United States; ⊥ Department of Biochemistry, Vanderbilt University, Nashville, Tennessee 37232, United States; # Center for Artificial Intelligence in Protein Dynamics, Vanderbilt University School of Medicine, Nashville, Tennessee 37232, United States; ¶ Department of Chemistry, Vanderbilt University, Nashville, Tennessee 37232, United States; ∇ Vanderbilt Kennedy Center, Vanderbilt University School of Medicine, Nashville, Tennessee 37232, United States; ○ Vanderbilt Brain Institute, Vanderbilt University School of Medicine, Nashville, Tennessee 37232, United States

**Keywords:** Metabotropic Glutamate Receptor Subtype 8, mGlu_8_, positive allosteric modulator (PAM), Parkinson’s disease, haloperidol-induced catalepsy, VU6081679

## Abstract

Herein, we report a potent, CNS penetrant, orally bioavailable metabotropic glutamate receptor subtype 8 (mGlu_8_) positive allosteric modulator (PAM), VU6081679, that shows robust efficacy in a rodent model of Parkinson’s disease. A high-throughput screening effort and subsequent structure–activity relationship study led to the discovery of VU6081679, an mGlu_8_-preferring PAM with good potency at rat, mouse, and human mGlu_8_. Molecular docking into a published cryo-EM structure supports binding in an allosteric pocket at the extracellular TM6/TM7 dimer interface. VU6081679 displays a favorable rodent DMPK profile and demonstrates robust efficacy in a haloperidol-induced catalepsy model of Parkinson’s disease in both rats and mice. Additional studies conducted in mGlu_8_ knockout mice confirmed that the observed efficacy is mGlu_8_-mediated.

## Graphical abstract

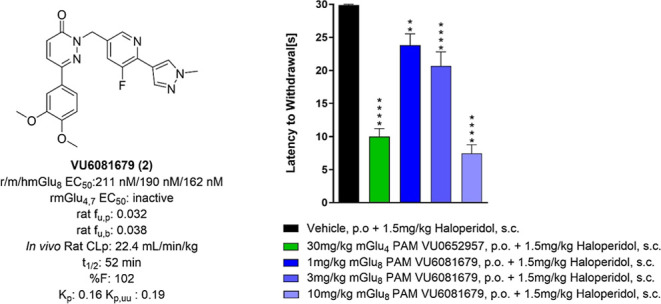

## Introduction

Parkinson’s disease (PD) is a debilitating neurodegenerative disease affecting more than 6 million people worldwide.^1^ Loss of dopaminergic neurons in the substantia nigra pars compacta located within the basal ganglia circuitry leads to the classical motor symptoms associated with PD, including bradykinesia, resting tremor, postural instability and rigidity.^2^ To date, there is no cure or disease-modifying therapeutic approach for PD patients, only symptomatic treatments focused on dopamine replacement therapies such as the dopamine precursor Levodopa (l-DOPA). Unfortunately, l-DOPA becomes less effective over time and with prolonged use is associated with the onset of dyskinesias and motor fluctuations (on–off periods).^3^

One nondopaminergic therapeutic approach studied by our group and others is the development of compounds that modulate key metabotropic glutamate (mGlu) receptor subtypes that are expressed in critical circuitry of the basal ganglia, particularly mGlu_4_.^4–17^ l-Glutamate (glutamate) modulates the activity of the mGlu receptors and serves as the primary excitatory neurotransmitter in the mammalian central nervous system. The eight known mGlu receptors (mGlu_1–8_) are family C G-protein-coupled receptors that are grouped based on sequence homology, pharmacology, and downstream signaling partners.^18^ Group I is composed of mGlu_1_ and mGlu_5,_ Group II is composed of mGlu_2_ and mGlu_3_ and Group III includes mGlu_4_, mGlu_6_, mGlu_7_, and mGlu_8_. The Group III mGlus are expressed presynaptically and couple to G_i/o_ G proteins, regulating cAMP levels and ion channels to modulate neurotransmitter release.^19^ Structurally, all eight mGlus exhibit a large extracellular amino-terminal domain (Venus fly trap, VFT), a cysteine-rich portion and a seven-transmembrane spanning (7TM) domain.^18,19^

The potential of mGlu_4_ activation or potentiation to treat PD was first discovered using the Group III mGlu receptor selective agonist, L-2-amino-4-phosphonobutyric acid (L-AP4, Figure 1). Application of L-AP4 reduces GABA release from terminals projecting from the striatum to the external segment of the globus pallidus, an effect that is lost in mGlu_4_ knockout mice.^20^ Intracerebroventricular injection of L-AP4 reverses motor defects in several rodent Parkinson’s models, including a haloperidol-induced catalepsy (HIC) model, reserpine-induced akinesia, and 6-hydroxydopamine lesions in rats.^20^ As agonists such as L-AP4 bind to a highly conserved orthosteric site in the VFT, achieving subtype selectivity is challenging. Allosteric modulation allows an approach for developing pharmacological tools or drugs with improved selectivity across the eight subtypes, and most known allosteric modulators bind in the 7TM domain.^21^ Additionally, true allosteric modulators are only active in the presence of the endogenous ligand and thus may have improved safety profiles due to the potential for modulatory control.^22^ (−)-*N*-Phenyl-7-(hydroxyimino)­cyclopropa­[*b*]­chromen-*1a*-carboxamide (PHCCC, Figure 1) was the first mGlu_4_ positive allosteric modulator (PAM) to be discovered and established potential for allosteric modulation of mGlu_4_ in Parkinson’s disease.^5,15^ Numerous mGlu_4_ PAMs from multiple laboratories have now shown efficacy in rodent and nonhuman primate PD models suggesting utility of this approach.^4,7,9,11,13,16,17,23^ Two advanced PAMs, Foliglurax and Valiglurax have been evaluated as preclinical development candidates for the treatment of PD.^12,13^ Valiglurax was not advanced due to low solubility, leading to a high projected human dose.^24^ Foliglurax entered clinical development and, although it was found to be safe and tolerated, it did not show efficacy in improving PD symptoms at the doses tested resulting in discontinuation of the clinical trials.^13,25–27^ More recently, another novel mGlu_4_ PAM clinical candidate, AP-472, developed by Appello Pharmaceuticals, has progressed into Phase II clinical testing in PD patients.^28^

**Figure 1:**
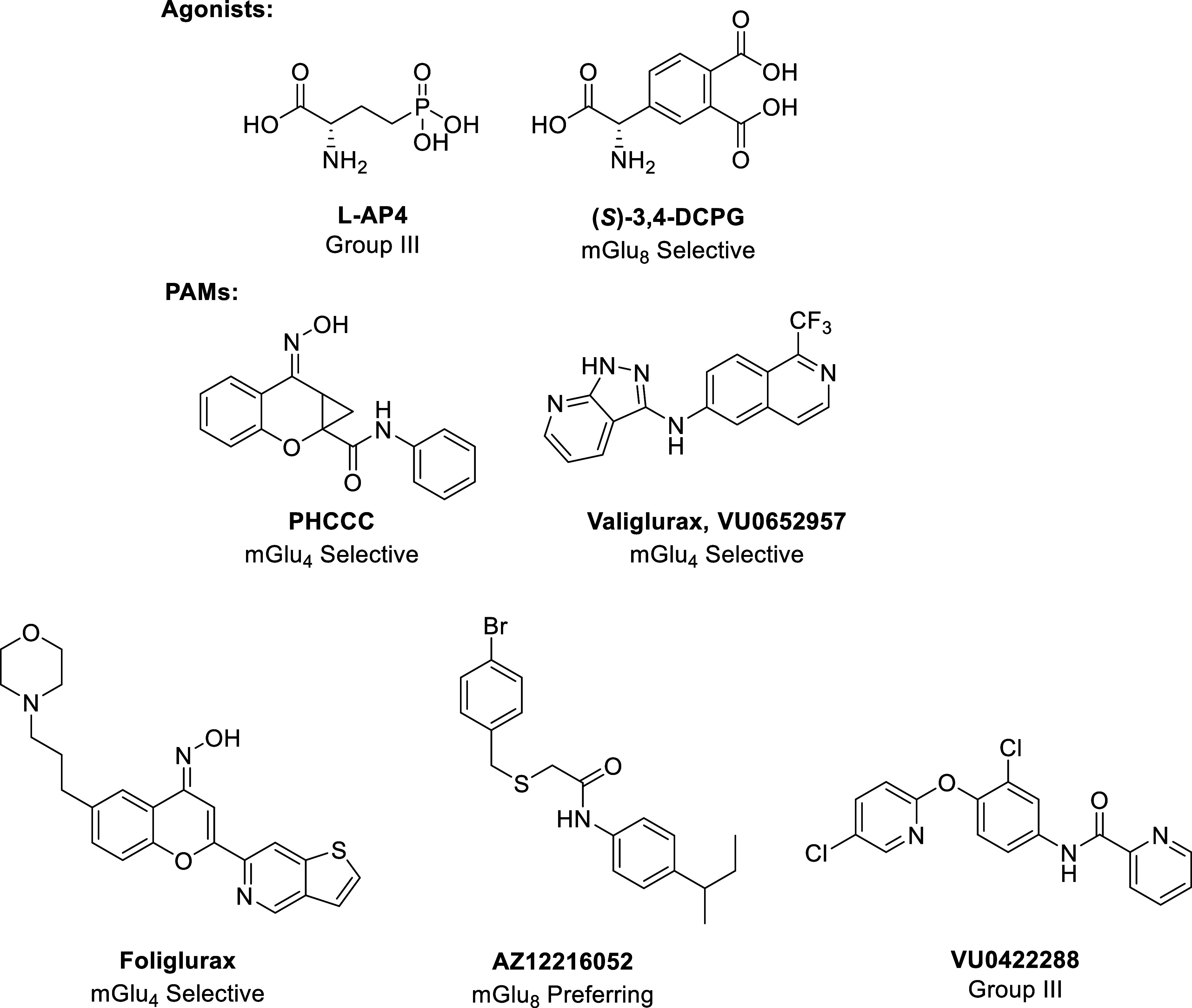
Group III mGlu Modulators.

While activation of mGlu_4_ is a well-established approach to modulate neurotransmission in the basal ganglia resulting in antiparkinsonian effects, another Group III mGlu receptor subtype, mGlu_8_, has been speculated to also play an important role in modulating basal ganglia circuitry relevant to PD. We previously reported that intracerebroventricular administration of (*S*)-3,4-dicarboxyphenylglycine ((*S*)-3,4-DCPG, Figure 1), an mGlu_8_ selective agonist reversed the effect of the D_2_ dopamine receptor antagonist haloperidol to induce catalepsy after a 24 h haloperidol treatment paradigm or using the long acting version of haloperidol, Haldec.^29,30^ (*S*)-3,4-DCPG also attenuated reserpine-induced akinsesia, a rodent PD model in which dopamine was depleted for more than 24 h. Collectively, these data provided early evidence that activation of mGlu_8_ may be a viable approach for treating motor symptoms associated with PD. Interestingly, in our study (*S*)-3,4-DCPG was ineffective in reversing HIC after acute dosing, suggesting that mGlu_8_ expression may change with dopamine depletion.^30^

AZ12216052 (Figure 1) has been reported as a selective mGlu_8_ PAM that reduces measures of anxiety in mice; however, in subsequent studies the compound also showed efficacy in mGlu_8_ knockout mice, leading the authors to conclude that other receptors were also being targeted.^31,32^ Clearly, additional potent and selective tool compounds are needed to further evaluate the therapeutic potential of mGlu_8_. We describe here the discovery of a potent, CNS penetrant mGlu_8_ PAM derived from a high-throughput screen (HTS) campaign hit that showed efficacy in an acute HIC model in both mice and rats; importantly, efficacy was lost in mGlu_8_ knockout mice. This work establishes the therapeutic value of positive allosteric modulation of mGlu_8_ for PD as a complement to mGlu_4_ and provides the scientific community with a rodent tool compound to further study the role mGlu_8_ may play as a therapeutic target for PD as well as other clinical disorders.

### Discovery of VU6081679

An HTS campaign of over 25,000 compounds utilized a calcium mobilization assay with cells expressing the human mGlu_8_ receptor and was performed with multiple compound and agonist additions to simultaneously identify mGlu_8_ agonists, PAMs, and NAMs as we have done previously.^33,34^ The screen successfully identified 822 PAM hits, and confirmation of mGlu_8_ activity and counter-screening against untransfected cells ultimately identified a series of pyridazinones represented by **1** (VU0626660, Figure 2) as an attractive lead series. A full concentration–response curve was generated and **1** displayed an EC_50_ of 942 nM at rat mGlu_8_ (47% maximal response compared to glutamate (% Glu Max)) in the presence of an EC_20_ concentration of glutamate. No effect of the compound was observed in the absence of glutamate in this assay. We next investigated the selectivity across other Group III mGlu receptors, and **1** was inactive on both the widely expressed and related mGlu_4_ and mGlu_7_ receptors (no activity at 30 μM). Compound **1** was next advanced into solubility and *in vitro* DMPK assays. Unfortunately, the compound suffered from low kinetic solubility (<1.2 μM at both pH = 2.2 and 6.8), high predicted rat clearance (>50 mL/min/kg), and high protein binding in rat plasma (<0.01 unbound).

**Figure 2:**
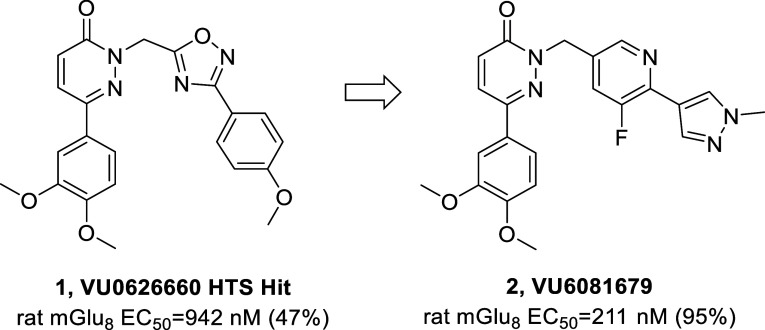
Following SAR optimization, HTS Hit, 1 (VU0626660), led to the discovery of mGlu_8_ PAM, VU6081679, 2.

We initiated a structure activity-relationship (SAR) campaign to identify additional selective mGlu_8_ PAMs with improved potency and DMPK properties. These efforts led to the discovery of VU6081679, **2**, a compound displaying an EC_50_ value of 211 nM at rat mGlu_8_ (95% Glu Max) (Figure 2). Additional details of these SAR studies will be reported in due course. During the course of SAR optimization, cryo-EM structures of mGlu_8_ became available, including a G_i_-bound active-state receptor dimer in complex with the Group III mGlu receptor PAM VU0422288 (PDB: 9MB9).^35,36^ These structures revealed an allosteric pocket at the extracellular TM6/TM7 dimer interface. To evaluate whether **2** may exert its PAM activity through engagement with this site, we performed flexible docking into the PAM-binding pocket of the G_i_-coupled mGlu_8_ dimer. Docking placed **2** in the intersubunit PAM site, overlapping the position of the cocrystallized mGlu_8_ PAM (Figure 3A). In the predicted pose, the dimethoxyphenyl ring is buried within a hydrophobic cleft formed by L795, A796, F797, I798, V820, and S821 on TM6 and TM7 at the dimer interface, with the *para*-methoxy group packed against the hydrophobic floor of the pocket. The heteroaryl portion of the ligand, including the methylpyrazole, extends toward the extracellular mouth of the pocket, where it is flanked by W583, I813, and F802 (Figure 3A).

**Figure 3:**
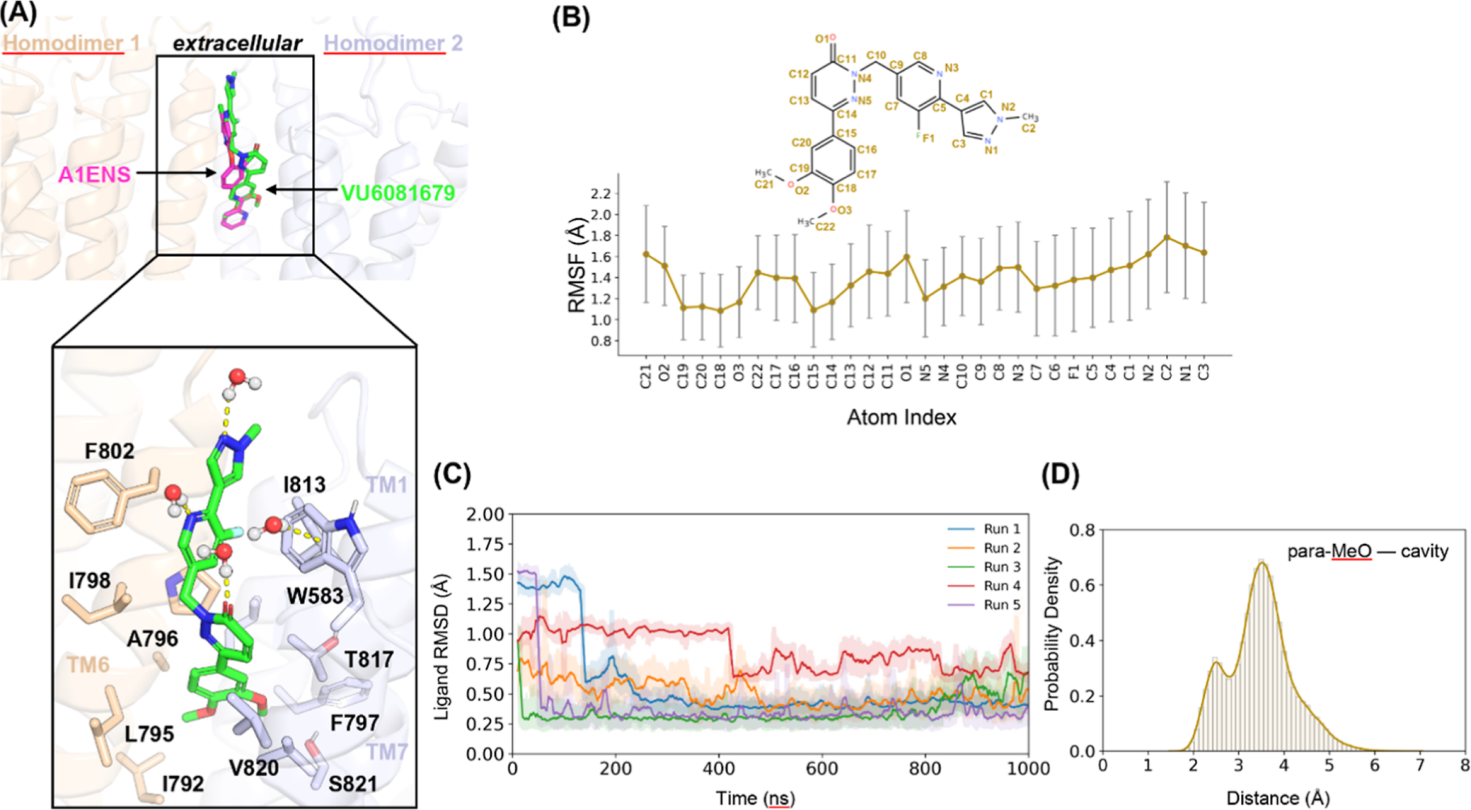
Docking and molecular dynamics simulations of VU6081679 binding to the mGlu_8_ PAM site. (A) Predicted binding mode of VU6081679 (green) in the extracellular intersubunit PAM pocket of the G_i_-coupled mGlu_8_ dimer, shown alongside the cocrystallized PAM (purple). The inset highlights the pocket environment: the dimethoxyphenyl group is buried among hydrophobic TM6/TM7 residues (L795, A796, F797, I798, V820, S821), while the heteroaryl scaffold extends toward the extracellular mouth, where structured water molecules (red spheres) mediate polar contacts with the ligand’s heterocyclic nitrogen atoms and nearby residues including W583, I813, and F802. Yellow dashed lines indicate polar contacts. (B) Per-atom RMSF of VU6081679 across five independent 1 μs MD simulations, mapped onto the ligand atom indexing scheme (inset). Error bars are standard deviation across the five independent trials. (C) Ligand RMSD relative to the average simulation pose over time for each replicate, showing stable retention of the docked binding mode. (D) Distance distribution between the *para*-methoxy group and the adjacent hydrophobic cavity wall, showing persistent van der Waals contact with populations centered near 2.5 and 3.5 Å.

To test the stability of this predicted binding mode in a membrane environment, we carried out five independent 1.0 μs all-atom molecular dynamics (MD) simulations of the membrane-embedded mGlu_8_ dimer in complex with **2**. Per-atom Root Mean Square Fluctuation (RMSF) analysis demonstrated relatively uniform flexibility across the ligand scaffold, with modestly elevated mobility at terminal atoms of the solvent-exposed methylpyrazole group (Figure 3B). Ligand Root Mean Square Deviation (RMSD) relative to the average simulation pose showed that **2** remained stably bound in the PAM pocket across replicate simulations (Figure 3C), and the *para*-methoxy group maintained persistent van der Waals contacts with the hydrophobic cavity wall throughout the simulations, as reflected by a bimodal distance distribution with populations centered near 2.5 and 3.5 Å (Figure 3D). We also noted persistent water densities on the extracellular side that form polar contacts with the heterocyclic nitrogen atoms and pyridazinone oxygen atom of **2**. These water molecules form bridging interactions with adjacent residues such as W583. Notably, the nitrogen in the pyridazinone is a buried unpaired hydrogen bond acceptor. Overall, this modeling suggests that **2** is anchored in the mGlu_8_ PAM pocket through burial of the dimethoxyphenyl group in the TM6/TM7 hydrophobic core, complemented by water-mediated polar contacts between the heterocyclic portion of the ligand and residues at the extracellular mouth of the intersubunit cavity.

### Molecular Pharmacology and *In Vitro* DMPK Profiling

This novel mGlu_8_ PAM, **2**, displayed potency agreement across species (rat EC_50_ = 211 nM, (95% Glu Max); mouse EC_50_ = 190 nM (53% Glu Max); human EC_50_ = 162 nM (59% Glu Max)) and was next assessed for mGlu selectivity (Figure 4). Compound **2** was selective over the other two widely CNS expressed Group III mGlus, mGlu_4_ and mGlu_7_, as well as the Group II mGlus, mGlu_2_ and mGlu_3_. The compound displayed weak PAM activity at the two Group I mGlu receptors (mGlu_1_ EC_50_ = 295 nM (42% Glu Max); mGlu_5_ EC_50_ = 1290 nM (49% Glu Max)). While efforts are currently underway to further improve selectivity, **2** was further advanced into a battery of *in vitro* DMPK assays to determine its potential as an *in vivo* tool compound (Tables 1 and 2).

**Figure 4:**
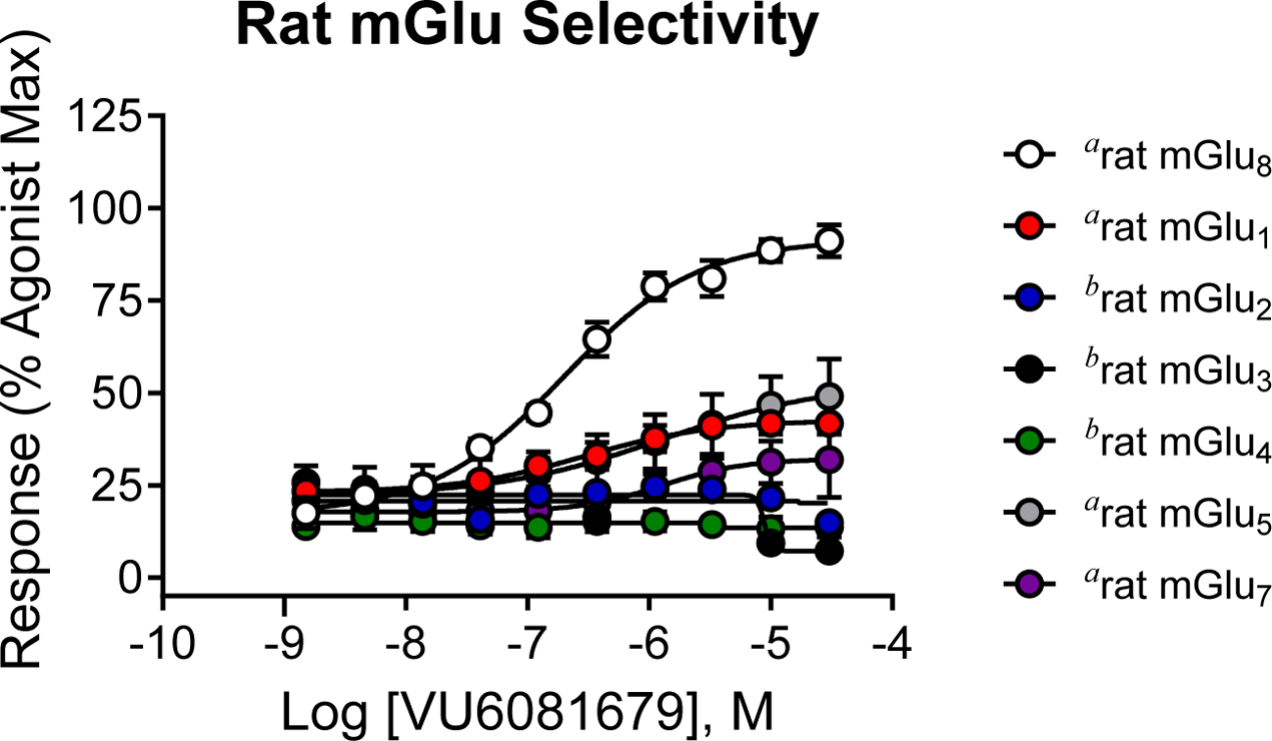
Metabotropic Glutamate Receptor Selectivity of 2. ^*a*^Calcium mobilization assay. ^*b*^Thallium flux through G protein inwardly rectifying potassium channels (GIRK) assay. Concentration–response curves are shown in the presence of a submaximal concentration that induces a ∼20% maximal response of agonist. Data are representative of one or more independent experiments run in duplicate or triplicate. Data are plotted as a percentage of maximal agonist response.

**Table 1:** *In Vitro* PK Profile of **2**

Property	**2**
	**VU6081679**
MW	421.16
*x*Log *P*	2.78
TPSA	81.1
*In Vitro* PK Parameters	
CL_int_ (mL/min/kg), rat	164
CL_hep_ (mL/min/kg), rat	49.1
CL_int_ (mL/min/kg), mouse	228
CL_hep_ (mL/min/kg), mouse	64.5
CL_int_ (mL/min/kg), human	75
CL_hep_ (mL/min/kg), human	16.4
rat *f*_*u*,plasma_ ^a^	0.032
rat *f*_*u*,brain_ ^a^	0.038
mouse *f*_*u*,plasma_ ^a^	0.088
mouse *f*_*u*,brain_ ^a^	0.044
human *f*_*u*,plasma_ ^a^	0.089
MDCK-MDR1 Permeability Assay	
P_app_ (A-B) (cm/s)	95.4 × 10^–6^
Efflux ratio	1.4
CYP450 Inhibition (IC_50_ in μM)	
1A2	>30
2C9	>30
2D6	>30
3A4	>30

a *f*_u_ = fraction unbound; equilibrium dialysis assay; brain = rat brain homogenates.

**Table 2:** *In Vivo* DMPK Profile of **2**

Property	**2**
	**VU6081679**
*In Vivo* PK Parameters^a^	
CL_p_ (mL/min/kg)	22.4
Elim. *t*_1/2_ (hr)	0.91
V_ss_ (L/kg)	1.38
%F	102
Brain Distribution^b^	
*K*_p brain/plasma_	0.16
K_p,uu brain/plasma_	0.19

a Male Sprague–Dawley rats (*n* = 2); IV PK: 1 mg/kg, vehicle = 10% Ethanol, 40% PEG400, 50% Saline; PO PK: 10 mg/kg, vehicle = 10% Tween80 in water.

b K_p_ = total brain-to-plasma partition ratio; K_p,uu_ = unbound brain-to-plasma partition ratio [(brain *f*_*u*_ × total brain)/(plasma *f*_*u*_ × total plasma)].

Both the molecular weight (421 g/mol) and cLogP (2.8) of **2** indicate favorable properties for a CNS drug. **2** displays acceptable kinetic solubility of 67.8 μM at pH = 2.2 and 13.5 μM at pH = 6.8. Predicted hepatic microsomal clearance was determined to be moderately high in rat (49.1 mL/min/kg), mouse (64.5 mL/min/kg) and human (16.4 mL/min/kg). The compound exhibited modest plasma free fraction in rat (*f*_u,plasma_ = 0.032) and high plasma free fraction in both mouse (*f*_u,plasma_ = 0.088) and human (*f*_u,plasma_ = 0.098) with satisfactory rodent brain homogenate binding in rat (*f*_u,brain_ = 0.038) and mouse (*f*_u,brain_ = 0.044). Brain penetration was assessed using MDCKII-MDR1 transfected cells. Compound **2** exhibited a *P*-glycoprotein (*P*-gp) efflux ratio (ER) of 1.4 and a P_app_ (A-B) of 95.4 × 10^–6^, indicating that it is not an efflux substrate. An ancillary pharmacology screen showed minimal off-target inhibition (Eurofins Panlabs; Supplemental Table 1). Additionally, the compound exhibited an excellent CYP450 profile as it does not inhibit any of the isoforms tested (IC_50_ > 30 μM at 1A2, 2C9, 2D6 and 3A4). These data are summarized in Table 1.

### *In Vivo* DMPK and Behavioral Pharmacology

To further evaluate the potential of **2** to serve as a rodent tool compound, rat IV/PO PK parameters were assessed (Table 2). In these studies, the compound demonstrated high oral bioavailability (>100%), moderate clearance (22.4 mL/min/kg), moderate volume of distribution (1.4 L/kg), and a moderate half-life (0.91 h). Total brain-to-plasma (*K*_p_) and unbound brain-to-plasma (*K*_p,uu_) ratios were determined to be 0.16 and 0.19, respectively, corresponding to moderate CNS penetration in rat. These data are summarized in Table 2. This is an overall attractive *in vivo* profile, though the moderate half-life may limit this tool compound to use in acute animal studies. This will be the subject of future optimization.

Based on the promising *in vivo* rat PK profile, we evaluated **2** in a rat haloperidol-induced catalepsy (HIC) assay, a preclinical model of motor symptoms associated with PD, in comparison with our preclinical mGlu_4_ PAM candidate, Valiglurax, as a positive control (Figure 5). Compound **2** exhibited a dose-dependent reversal of HIC in rats, with the 10 mg/kg dose showing similar efficacy to the 30 mg/kg dose of Valiglurax (VU0652957) (75.7% vs 66.7% reversal, respectively). The PK/PD relationship shows that, at the unbound brain concentration achieved at the 10 mg/kg dose, the exposure is 1.6 times the *in vitro* rat mGlu_8_ EC_50_ (Table 3).

**Figure 5:**
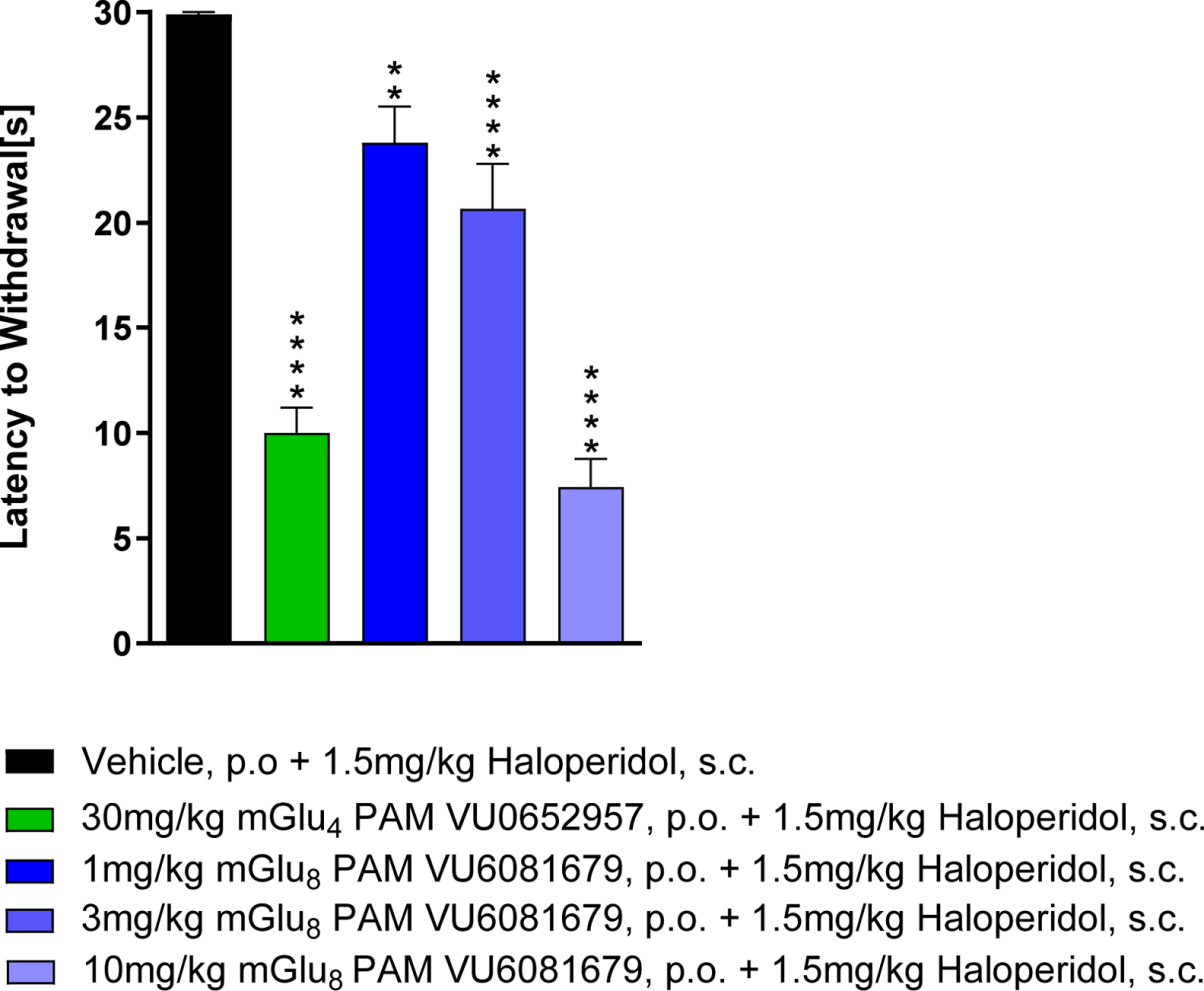
Orally administered **2** dose-dependently reversed haloperidol-induced catalepsy in rats. *N* = 6–14 rats per group; **p < 0.001, ****p < 0.0001 vs Vehicle + Haloperidol control; Vehicle = 10% Tween 80; VU0652957 = positive control.

**Table 3:** Exposure of VU6081697 (**2**) and VU0652957 in HIC Study

compound	dose (mg/kg)	% reversal ± SEM	plasma total (μM)	plasma free (nM)	brain total (μM)	brain free (nM)	fold EC_50_ (free brain)
VU6081679	1	20.7 ± 4.0	4.28	0.14	1.04	39.3	0.19
	3	31.1 ± 7.1	12.0	0.39	2.95	111	0.53
	10	75.7 ± 3.9	21.1	0.68	8.77	332	1.57
VU0652957	30	66.7 ± 4.0	3.52	0.06	6.14	181	0.92^a^

a To calculate the Fold EC_50_ value for VU0652957, the rat mGlu_4_ EC_50_ (197 nM) reported in Panarese et al., 2019 was used.

To confirm that the observed antiparkinsonian effects in rats were mGlu_8_-mediated, we evaluated the effects of **2** in the HIC model using both wild-type and mGlu_8_ knockout mice. For these studies, we generated a complete knockout of the mouse mGlu_8_ gene by crossing a floxed *Grm8* allele to CMV-Cre, which results in a knockout of mGlu_8_ in all tissue; the CMV-Cre allele was then eliminated by breeding.^37,38^ Our initial experiment was to provide documented confirmation that haloperidol induces a similar level of catalepsy in both the wild-type and mGlu_8_ knockout mice. As shown in Figure 6, both groups of mice exhibited a robust catalepsy response that was comparable across genotype 120 min post haloperidol (2.0 mg/kg i.p.) administration.

**Figure 6:**
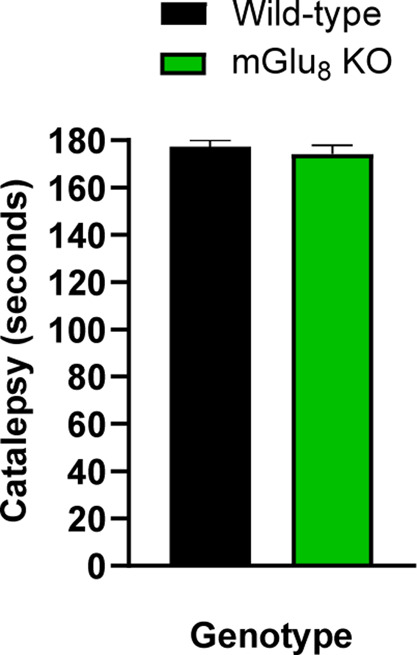
Wild-type and mGlu_8_ knockout mice showed robust catalepsy 120 min post haloperidol (2.0 mg/kg i.p.) administration. *N* = 6–9 mice per group Vehicle = Sterile water.

Based on these studies, we found that **2** reversed HIC in both male and female wild-type mice to a similar magnitude as previously observed in rats, but had no effect in mGlu_8_ knockout mice when assessed at 30 min after VU6081679 administration (Figure 7) and efficacy persisted until 90 min after administration (Supplemental Table 2). These results confirm that the antiparkinsonian effects of **2** are mGlu_8_-mediated. We note that these results in an acute HIC model are in contrast to our previous work with (*S*)-DCPG, where we did not observe an effect in models of acute changes in dopamine, as well as a report by Ossowska et al. in which (*S*)-DCPG actually enhanced catalepsy.^30,39^ An explanation for these discrepancies may lie in the ability of mGlu receptors to form heterodimers, which can dramatically impact receptor pharmacology.^9,40–45^ We have recently found that, when complexed into an mGlu_7/8_ heterodimer, (*S*)-DCPG no longer acts as an agonist; the compound still binds to the mGlu_8_ protomer and now blocks the activity of another orthosteric agonist, DL-AP4.^46^ Additional studies will be needed to address these different *in vivo* profiles of (*S*)-DCPG and **2**. Studies to isolate homodimer and heterodimer effects are in progress and will be reported in due course.

**Figure 7:**
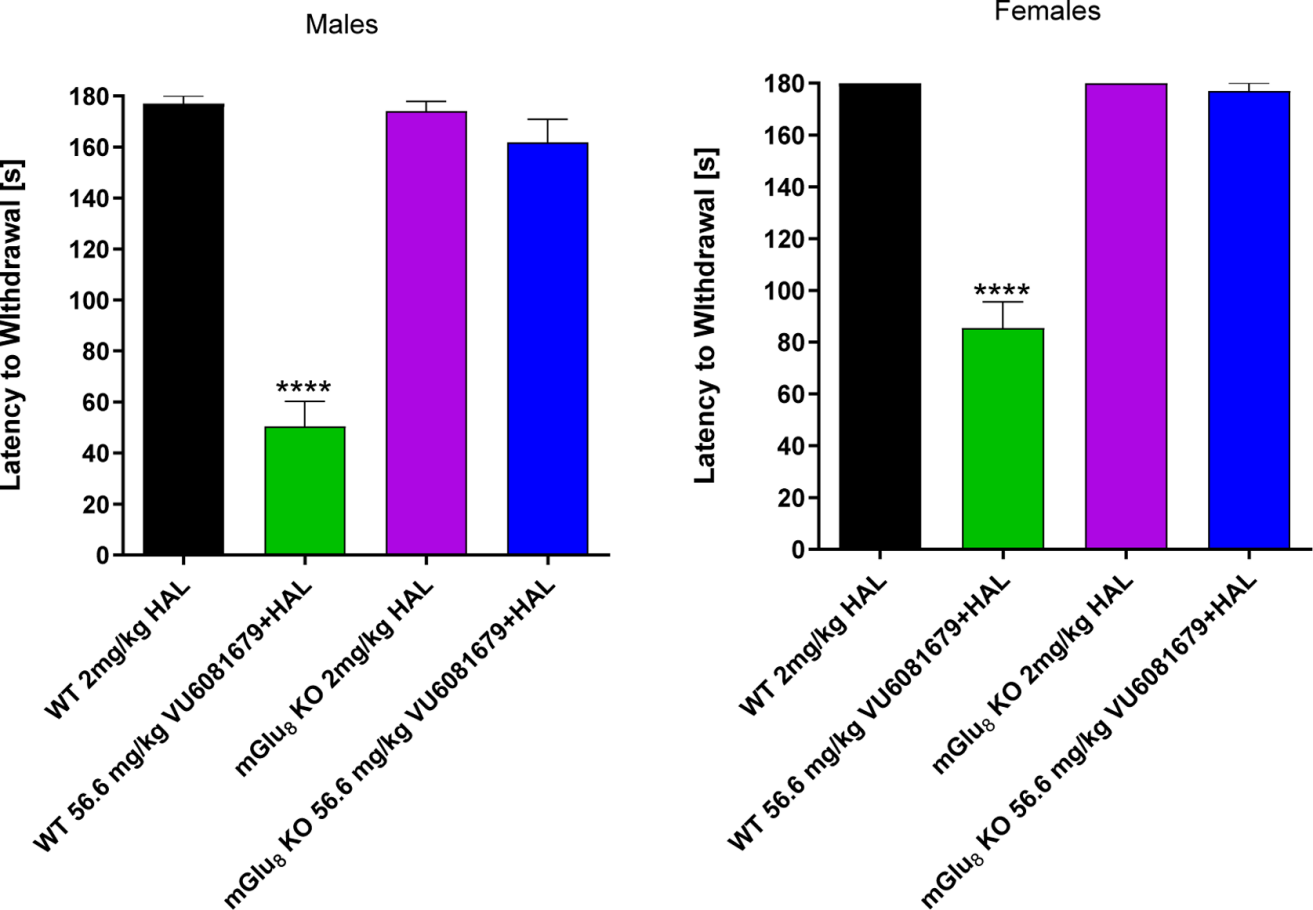
Intraperitoneally administered **2**, at a dose of 56.6 mg/kg, reversed haloperidol-induced catalepsy in wild-type mice, but not mGlu_8_ KO mice. Left graph: *N* = 4–5 Male mice per group. Right graph: *N* = 6–7 Female mice per group. ****p < 0.0001 vs Vehicle + Haloperidol control; Vehicle = 10% Tween 80.

## Conclusions

In summary, following an HTS campaign, structure activity relationship studies led to the discovery of **2**, **VU6081679**. **VU6081679** is a potent, CNS penetrant, positive allosteric modulator of metabotropic glutamate receptor subtype 8 which displays a good rodent PK profile, allowing for *in vivo* evaluation. The compound exhibited efficacy in an acute haloperidol-induced catalepsy model of Parkinson’s disease in two rodent species, and this effect was consistent with mGlu_8_ potentiation based on studies in mGlu_8_ knockout mice. This work provides proof-of-principle for the therapeutic potential of activation of mGlu_8_ in Parkinson’s disease and the discovery of **VU6081679** will allow further exploration of the role mGlu_8_ plays in both physiological and pathophysiological domains.

## Methods

### Synthesis

#### 6-(3,4-Dimethoxyphenyl)-2-((5-fluoro-6-(1-methyl-1*H*-pyrazol-4-yl)­pyridin-3-yl)­methyl)­pyridazin-3­(2*H*)-one (**2**)

To a solution of 6-(3,4-dimethoxyphenyl)­pyridazin-3­(2*H*)-one (2.3 g, 9.94 mmol) and 5-(chloromethyl)-3-fluoro-2-(1-methyl-1*H*-pyrazol-4-yl)­pyridine hydrochloride (2.9 g, 10.94 mmol) in DMF (100 mL) was added cesium carbonate (16 g, 49.71 mmol). After 18h at rt, water (1.4 L) was added and the mixture stirred for 10 min. The resulting solid was collected by vacuum filtration, sonicated in *iso*-propanol (50 mL), and again collected by vacuum filtration to afford the title compound (3.2 g, 75% yield). ^1^H NMR (400 MHz, DMSO-*d*_6_): δ 8.46 (t, *J* = 1.8 Hz, 1H), 8.27 (d, *J* = 2.2 Hz, 1H), 8.09 (d, *J* = 9.8 Hz, 1H), 7.96 (dd, *J* = 1.6, 0.7 Hz, 1H), 7.75 (dd, *J* = 11.8, 1.8 Hz, 1H), 7.45 (dd, *J* = 8.4, 2.1 Hz, 1H), 7.42 (d, *J* = 2.1 Hz, 1H), 7.07 (d, *J* = 9.8 Hz, 1H), 7.05 (d, *J* = 8.5 Hz, 1H), 5.37 (s, 2H), 3.91 (s, 3H), 3.81 (s, 3H), 3.80 (s, 3H). ^13^C NMR (101 MHz, DMSO-*d*_6_): δ 158.73, 156.01, 153.45, 150.13, 149.01, 145.47, 145.43, 143.88, 139.55, 139.42, 137.89, 137.84, 131.24, 131.21, 130.99, 130.82, 130.73, 129.81, 126.73, 123.81, 123.62, 118.82, 116.90, 116.84, 111.72, 108.98, 55.58, 55.51, 51.33, 38.71. HR-MS (Q-TOF, ES+) calcd for C_22_H_20_FN_5_O_3_, 422.1623; found, 422.1623.

### Molecular Pharmacology

#### mGlu_8_ Calcium Mobilization Assays

Human Embryonic Kidney 293 (HEK293 cells) stably coexpressing rat, mouse, or human mGlu_8_ and the promiscuous G protein G_α15_ (mouse) were maintained in DMEM containing 10% fetal bovine serum, 20 mM HEPES, 1 mM sodium pyruvate, 2 mM l-glutamine, nonessential amino acids mixture, 100 units/mL antibiotic/antimycotic, 700 μg/mL G418 sulfate, and 0.6 μg/mL puromycin. All cell culture reagents were purchased from Invitrogen (Carlsbad, CA) unless otherwise stated.

Rat, mouse, or human mGlu_8__G_α15__HEK cells (15,000 cells/20 μL/well for rat, 20,000 cells/20 μL/well for mouse and human) were plated in black-walled, clear-bottomed, poly-d-lysine coated, 384 well plates (Corning Inc., Corning, NY) in DMEM containing 10% dialyzed FBS, 20 mM HEPES, 100 units/mL antibiotic/antimycotic, and 1 mM sodium pyruvate. The cells were grown overnight at 37 °C in the presence of 5% CO_2_. The next day, calcium assay buffer (Hank’s balanced salt solution (HBSS), 20 mM HEPES, 2.5 mM Probenecid, 4.16 mM sodium bicarbonate (Sigma-Aldrich, St. Louis, MO)) was prepared to dilute compounds, agonists, and Fluo-4-acetomethoxyester (Fluo-4-AM, Ion Biosciences), a fluorescent calcium indicator dye. Compounds were serially diluted 1:3 into 10-point concentration response curves in DMSO using a Bravo Liquid Handler (Agilent, Santa Clara, CA), transferred to a 384 well daughter plate using an Echo acoustic liquid handler (Beckman Coulter, Indianapolis, IN), and diluted in assay buffer to a 2X final concentration. The agonist plates were prepared using glutamate (Tocris, Minneapolis, MN) concentrations for the EC_20_, EC_80_ and EC_Max_ responses by diluting in assay buffer to a 5X final concentration. A 2X dye solution (2.3 μM) was prepared by mixing a 2.3 mM Fluo-4-AM stock in DMSO with 10% (w/v) pluronic acid F-127 in a 1:1 ratio in assay buffer. Using a microplate washer (BioTek, Winooski, VT), cells were washed with assay buffer 3 times to remove media. After the final wash, 20 μL of assay buffer remained in the cell plates. Twenty μL of the 2X dye solution (final 1.15 μM) was added to each well of the cell plate using a Multidrop Combi dispenser (Thermo Fisher, Waltham, MA). After cells were incubated with the dye solutions for 45 min at 37 °C in the presence of 5% CO_2_, the dye solutions were removed and replaced with assay buffer using a microplate washer, leaving 20 μL of assay buffer in the cell plate. The plate was incubated for 10 min at 37 °C prior to the assay. The compound, agonist, and cell plates were placed inside the Functional Drug Screening System (FDSS 7000 or μCell, Hamamatsu, Japan) to measure the calcium flux. Assays were run at 37 °C. A triple add protocol was used to measure calcium kinetics. Briefly, after establishment of a fluorescence baseline for 2 s (excitation, 480 nm; emission, 530 nm, readings collected at 1 Hz), 20 μL of test compound was added to the cells and the response was measured for 142 s. This was followed by the addition of 10 μL (5X) of an EC_20_ concentration of glutamate agonist, and the response of the cells was measured for 125 s. A third addition occurred by adding 12 μL (5X) of an EC_80_ concentration of glutamate agonist and the response of the cells was measured for 90 s. Vehicle (0.6% DMSO, 2x the final concentration) in assay buffer was added to the control wells at the first add to ensure vehicle matching for the glutamate EC_20_, EC_80_, and EC_Max_ additions. Calcium fluorescence was recorded as fold over basal fluorescence and raw data were normalized to the maximal response to glutamate agonist (EC_Max_). Potency (EC_50_) and maximum response (% Glutamate Max) for compounds were determined using a four-parameter logistical equation in GraphPad Prism (La Jolla, CA) or the Dotmatics software platform (Dotmatics, Bishop’s Stortford, UK).

$$y=bottom+\frac{top-bottom}{1+10^{\left(\log EC50-A\right)Hillslope}}$$

where *A* is the molar concentration of the compound; bottom and top denote the lower and upper plateaus of the concentration–response curve; Hillslope is the Hill coefficient that describes the steepness of the curve; and EC_50_ is the molar concentration of compound required to generate a response halfway between the top and bottom.

### Behavioral Pharmacology Methods

#### Rats

Male Sprague–Dawley rats weighing between 320 and 360 g (ENVIGO, Inc. Indianapolis, IN) were used for the behavioral studies and were housed maintained under a 12 h light/dark cycle (lights on at 6 AM, lights off at 6 PM) with free access to food and water. The experimental protocols, which were performed during the light cycle, were approved by the Institutional Animal Care and Use Committee of Vanderbilt University and conformed to the guidelines established by the National Research Council Guide for the Care and Use of Laboratory Animals (IACUC protocol #M2400066–00 and M2400056-00).

#### Mice

Global mGlu_8_ KO mice and wild-type (WT) littermates 12–15 weeks of age were used for this study. These mice were derived from homozygous *Grm8* floxed mice generously gifted by the laboratory of Dr. Danny Winder and previously validated for genetic deletion of mGlu_8_ using Cre-Lox recombination.^38^ To generate global mGlu_8_ KO mice, homozygous *Grm8* floxed mice were crossed to a germline Cre recombinase-expressing mouse line (B6.C-Tg­(CMV-cre)­1Cgn/J; Jax strain #006054) to achieve heterozygous deletion of exon 3 of the *Grm8* gene with hemizygous CMV-Cre expression in offspring. Heterozygous mGlu_8_ KO mice were bred over subsequent generations to produce offspring expressing the heritable recombined *Grm8* KO allele. Homozygous recombined *Grm8* KO mice were then crossed to Jax WT C57Bl6/J mice (Jax strain #000664), and offspring lacking CMV-Cre expression but retaining heterozygous recombined *Grm8* KO allele expression were then used as breeders for the experiments in this study. Thus, the mGlu_8_ KO mice used in this study did not contain the CMV-Cre allele to avoid potentially confounding behavioral phenotypes caused by germline expression of Cre-recombinase. Genotyping for mice was performed via qPCR by Transnetyx genotyping services.

#### Catalepsy

Rats were pretreated with haloperidol lactate (1.5 mg/kg, s.c., dissolved in sterile water) with vehicle (10% Tween 80) or VU6081679 (1–10 mg/k, p.o.) was administered 30 min later, In the case of the comparator compound mGlu_4_ PAM VU0652957, a 30 mg/kg dose, p.o., was administered 60 min after haloperidol administration. Rat were assessed for catalepsy 120 min posthaloperidol administration. Catalepsy was measured by placing the forepaws of each rat gently onto a horizontal bar placed 6 cm from the testing surface with the body positioned at an angle of ∼45° to the testing surface. The latency in seconds required for the rat to remove one or both forepaws from the bar was measured with a testing cutoff of 30 s.

Mice were administered with haloperidol lactate (2 mg/kg, dissolved in sterile water, i.p.) then administered either vehicle (10% Tween 80) or VU6081679 (56.6 mg/k, i.p.) 90 min later. Catalepsy was assessed 120 min posthaloperidol administration. Catalepsy was measured by placing the forepaws of each rat gently onto a horizontal bar placed 3 cm from the testing surface and with the body positioned at an angle of ∼45° to the testing surface. The latency in seconds required for the mice to remove one or both forepaws from the bar was measured with a testing cutoff of 180 s.

Catalepsy latency was analyzed by One-Way ANOVA followed by Dunnett’s posthoc analysis (GraphPad Prism 10 [GraphPad Software, San Diego, CA]). For all tests, *p* ≤ 0.05 was considered to represent statistical significance. Finally, percent reversal data were calculated in Microsoft Excel using the following formula: Percent Reversal = 100 – {[(Catalepsy in seconds in treated individual animal/(mean catalepsy in seconds Vehicle + Halopeidol group)] * 100}. Mean percent reversal ± SEM was calculated for each dose group using GraphPad Prism 10.

#### Sample Collection

To determine the plasma and brain levels of VU6081679 in the presence of haloperidol, brain and trunk blood samples were collected 120 min post haloperidol treatment. Plasma was collected via centrifugation (17,000 rpm, 5 min, 4 °C) and placed on wet ice. Whole brain samples were obtained by rapid dissection, rinsed with saline, and immediately frozen in individual tissue collection box (dry ice). All brain and plasma samples were stored at −80 °Cuntil analysis by LC–MS/MS.

## Supplementary Material



## Notes

General methods for the synthesis and characterization of intermediates, molecular modeling methods, experimental details for all assays performed (mGlu Selectivity, DMPK, additional ancillary pharmacology data) (PDF)

## Abbreviations

CNS: central nervous system
CYP450: cytochrome P450
DCPG: (S)-3,4-dicarboxyphenylglycine
DMPK: drug metabolism and pharmacokinetics
ER: efflux ratio
GIRK: G protein inwardly rectifying potassium channel
HIC: haloperidol-induced catalepsy
HTS: high-throughput screen
K_p_: total brain to plasma ratio
L-AP4: L-2-amino-4-phosphonobutyric acid
l-DOPA: levodopa
MD: molecular dynamics
mGlu: metabotropic glutamate receptor
PAM: positive allosteric modulator
PD: Parkinson’s disease
*P*-gp: p-glycoprotein
RMSD: Root Mean Square Deviation
RMSF: Root Mean Square Fluctuation
SAR: structure–activity relationship
TM: transmembrane
