# Supplementary material for: Discovery of VU6081679, a Novel Positive Allosteric Modulator of Metabotropic Glutamate Receptor 8 with Efficacy in a Preclinical Model of Parkinson’s Disease
Source: ACS Chem Neurosci. 2026 Jul 16;17(15):3023–34. doi: 10.1021/acschemneuro.6c00364 (PMC13449765; doi:10.1021/acschemneuro.6c00364)
Supplement: Supplementary file 1 [file cn6c00364_si_001.pdf]

Discovery of VU6081679, a novel positive allosteric modulator of metabotropic glutamate receptor 8 with efficacy in a preclinical model of Parkinson's disease

Alexa E. Richardson[a,b], Anthony S. Ferranti[a,b,c], Li Peng[a,b,c], Xia Lei[a,b,c], Allie Han[a,b,c], Aidong Qi[a,b,c], Alice L. Rodriguez[a,b,c], Sichen Chang[a,b,c], Snehal Sant[a,b,c], Irene Zagol-Ikapitte[a,b,c], Srinivasan Krishnan[a,b,c], Analisa Thompson Gray[a,b,c], Joshua C. Wilkinson[a,b,c], Sri H. Kethanapalli[a,b,c], Hyekyung P. Cho[a,b,c], Kayla J. Temple[a,b,c], Dehui Mi[d], Emily L. Days[d], Joshua A. Bauer[d,e], N. Kithmini Wijesiri[b,f], Benjamin P. Brown[b,f], Olivier Boutaud[a,b,c], Carrie K. Jones[a,b,c], Darren W. Engers[a,b,c], Craig W. Lindsley[a,b,c,e,g], Colleen M. Niswender[a,b,c,h,i]*, Katherine E. Crocker[a,b,c]*

[a]Warren Center for Neuroscience Drug Discovery, Vanderbilt University, Nashville, TN 37232, USA
[b]Department of Pharmacology, Vanderbilt University School of Medicine, Nashville, TN 37232, USA
[c]Vanderbilt Institute for Therapeutic Advances, Vanderbilt University School of Medicine, Nashville, TN 37232, USA
[d]Vanderbilt Institute of Chemical Biology, Vanderbilt University, Nashville, TN 37232, USA
[e]Department of Biochemistry, Vanderbilt University, Nashville, TN 37232, USA
[f]Center for Artificial Intelligence in Protein Dynamics, Vanderbilt University School of Medicine, Nashville, TN 37232, USA
[g]Department of Chemistry, Vanderbilt University, Nashville, TN 37232, USA
[h]Vanderbilt Kennedy Center, Vanderbilt University School of Medicine, Nashville, TN 37232, USA
[i]Vanderbilt Brain Institute, Vanderbilt University School of Medicine, Nashville, TN 37232, USA

*To whom correspondence should be addressed at katherine.crocker@vanderbilt.edu and colleen.niswender@vanderbilt.edu

Table of Contents

Synthesis 2
General Information 2
Synthesis of Intermediates 4
NMR Spectra of VU6081679 (2) 7
Eurofins Lead Profiling Screen 8
Supplemental Table S1. Eurofins Lead Profiling Screen Data-Study FR095-0054028[1] 8
Molecular Modeling 10
mGlu Selectivity Assays 11
DMPK Methods 12
Haloperidol-Induced Catalepsy in Mice 19
Supplemental Table 2. Time dependent effects of 2 in WT and $mGlu_8$ KO mice 19
References 19

Synthesis

General Information

All reactions were carried out employing standard chemical techniques. Solvents used for extraction, washing, and chromatography were HPLC grade. All reagents were purchased from commercial sources and were used without further purification.

Automated flash column chromatography was performed on a Biotage Isolera 1 or a Teledyne ISCO CombiFlash® R*f*+ system. RP-HPLC was performed on a Gilson preparative reversed-phase HPLC system comprised of a 333 aqueous pump with solvent-selection valve, 334 organic pump, GX-271 or GX-281 liquid hander, two column switching valves, and a 155 UV detector. Absorbance was typically monitored at 215 or 220 nm. Column: Phenomenex Axia-packed Gemini C18, 5 µm. Mobile phase: CH_3CN in H_2O (0.1% TFA) or CH_3CN in H_2O (0.05% *v/v* NH_4OH) under the specified gradient, then hold 95% CH_3CN in 5% aqueous phase, 50 mL/min, 23° C. All compounds were found to be >95% pure by LCMS analysis.

***Safety statement**: no unexpected or unusually high safety hazards were encountered.*

All NMR spectra were recorded on a 400 MHz AMX Bruker NMR spectrometer. ^1H and ^{13}C chemical shifts are reported in δ values in ppm downfield with the deuterated solvent as the internal standard. Data are reported as follows: chemical shift, multiplicity (s = singlet, d = doublet, t = triplet, q = quartet, b = broad, m = multiplet), integration, coupling constant (Hz).

Low resolution mass spectra (LRMS) were obtained on an Agilent 6120/6150 or Waters QDa (Performance) SQ MS with ESI source. *Method A (Agilent 6120/6150):* MS parameters were as follows: fragmentor: 70, capillary voltage: 3000 V, nebulizer pressure: 30 psig, drying gas flow: 13 L/min, drying gas temperature: 350 ºC. Samples were introduced via an Agilent 1290 UHPLC comprised of a G4220A binary pump, G4226A ALS, G1316C TCC, and G4212A DAD with ULD flow cell. UV absorption was generally observed at 215 nm and 254 nm with a 4 nm bandwidth. Column: Waters Acquity BEH C18, 1.0 x 50 mm, 1.7 um. Gradient conditions: 5% to 95% CH_3CN in H_2O (0.1% TFA) over 1.4 min, hold at 95% CH_3CN for 0.1 min, 0.5 mL/min, 55 ºC. *Method B (Agilent 6120/6150):* MS parameters were as follows: fragmentor: 100, capillary voltage: 3000 V, nebulizer pressure: 40 psig, drying gas flow: 11 L/min, drying gas temperature: 350 ºC.

Samples were introduced via an Agilent 1200 HPLC comprised of a degasser, G1312A binary pump, G1367B HP-ALS, G1316A TCC, G1315D DAD, and a Varian 380 ELSD (if applicable). UV absorption was generally observed at 215 nm and 254 nm with a 4 nm bandwidth. Column: Thermo Accucore C18, 2.1 x 30 mm, 2.6 um. Gradient conditions: 7% to 95% CH_3CN in H_2O (0.1% TFA) over 1.6 min, hold at 95% CH_3CN for 0.35 min, 1.5 mL/min, 45 ºC. *Method C (Waters QDa (Performance) SQ MS)*: MS parameters were as follows: cone voltage: 15 V, capillary voltage: 0.8 kV, probe temperature: 600° C. Samples were introduced via an Acquity I-Class PLUS UPLC comprised of a BSM, FL-SM, CH-A, and PDA. UV absorption was generally observed at 215 nm and 254 nm; 4 nm bandwidth. Column: Phenomenex EVO C18, 1.0 x 50 mm, 1.7 um. Column temperature: 55° C. Flow rate: 0.4 mL/min. Default gradient: 5% to 95% CH_3CN (0.05% TFA) in H_2O (0.05% TFA) over 1.4 min (curve 6), hold at 95% CH_3CN for 0.1 min. "Polar" (2% to 70% CH_3CN (0.05% TFA) in H_2O (0.05% TFA) over 0.8 min (curve 6), transition to 95% CH_3CN over 0.1 min (curve 6), hold at 95% CH_3CN for 0.6 min.) and "Non-Polar" (40% to 95% CH_3CN (0.05% TFA) in H_2O (0.05% TFA) over 1.4 min (curve 6), hold at 95% CH_3CN for 0.1 min.) gradients were also available. *Method D (Waters QDa (Performance) SQ MS):* MS parameters were as follows: cone voltage: 15 V, capillary voltage: 0.8 kV, probe temperature: 600° C. Samples were introduced via an Acquity I-Class PLUS UPLC comprised of a BSM, FL-SM, CH-A, and PDA. UV absorption was generally observed at 215 nm and 254 nm with a 4 nm bandwidth. Column: Phenomenex EVO C18, 1.0 x 50 mm, 1.7 um. Column temperature: 55° C. Flow rate: 0.4 mL/min. Default gradient: 5% to 95% CH_3CN in H_2O (5 mM NH_4HCO_3) over 1.4 min (curve 6), hold at 95% CH_3CN for 0.1 min. "Polar" (2% to 70% CH_3CN in H_2O (5 mM NH_4HCO_3) over 0.8 min (curve 6), transition to 95% CH_3CN over 0.1 min (curve 6), hold at 95% CH_3CN for 0.6 min.) and "Non-Polar" (40% to 95% CH_3CN in H_2O (5 mM NH_4HCO_3) over 1.4 min (curve 6), hold at 95% CH_3CN for 0.1 min.) gradients were also available.

High resolution mass spectra (HRMS) were obtained on an Agilent 6540 UHD Q-TOF with ESI source. MS parameters were as follows: fragmentor: 150, capillary voltage: 3500 V, nebulizer pressure: 60 psig, drying gas flow: 13 L/min, drying gas temperature: 275 ºC. Samples were introduced via an Agilent 1200 UHPLC comprised of a G4220A binary pump, G4226A 3 ALS, G1316C TCC, and G4212A DAD with ULD flow cell. UV absorption was observed at 215 nm and 254 nm with a 4 nm bandwidth. Column: Agilent Zorbax Extend C18, 1.8 μm, 2.1 x 50 mm.

Gradient conditions: 5% to 95% CH_3CN in H_2O (0.1% formic acid) over 1 min, hold at 95% CH_3CN for 0.1 min, 0.5 mL/min, 40 ºC.

Synthesis of Intermediates

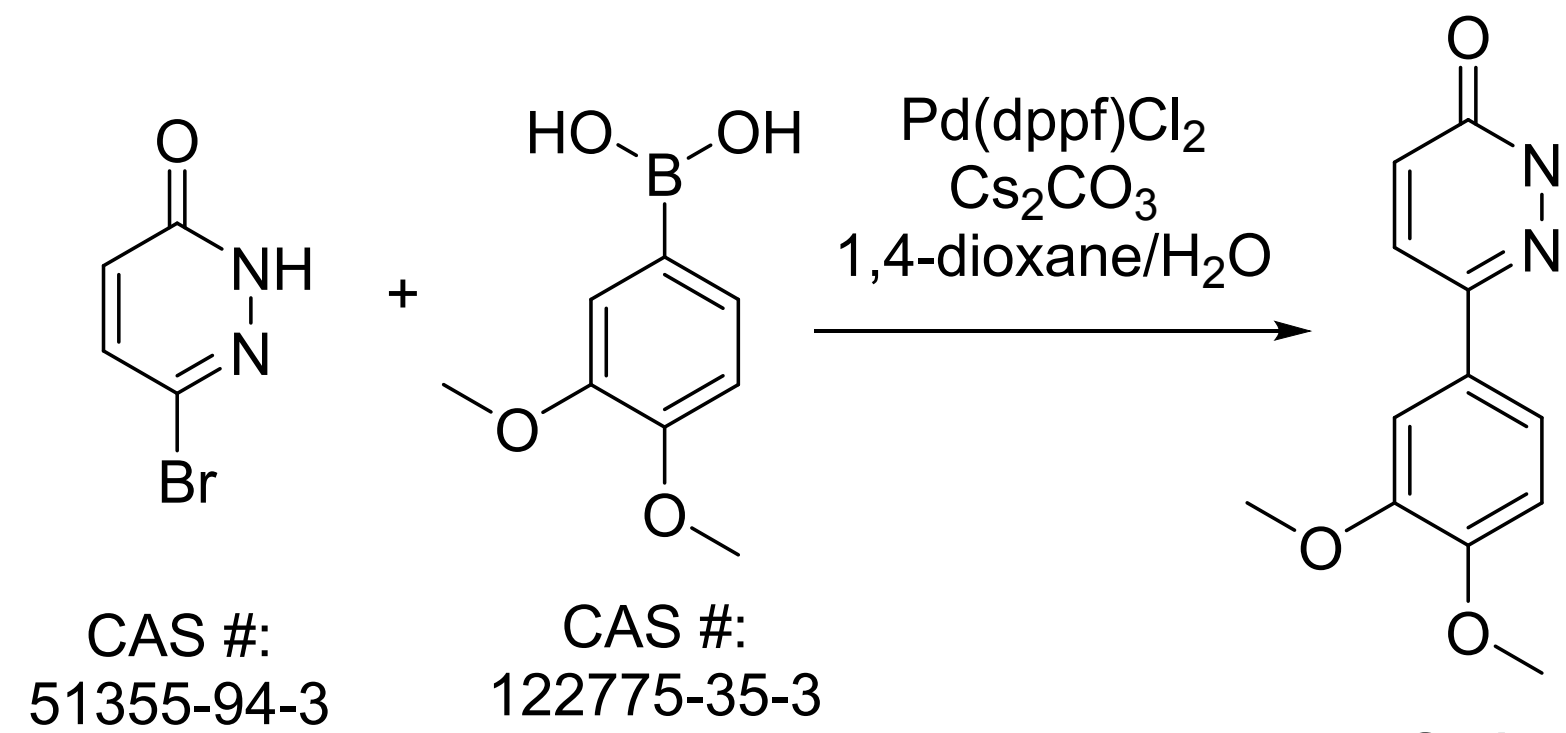

SI-1

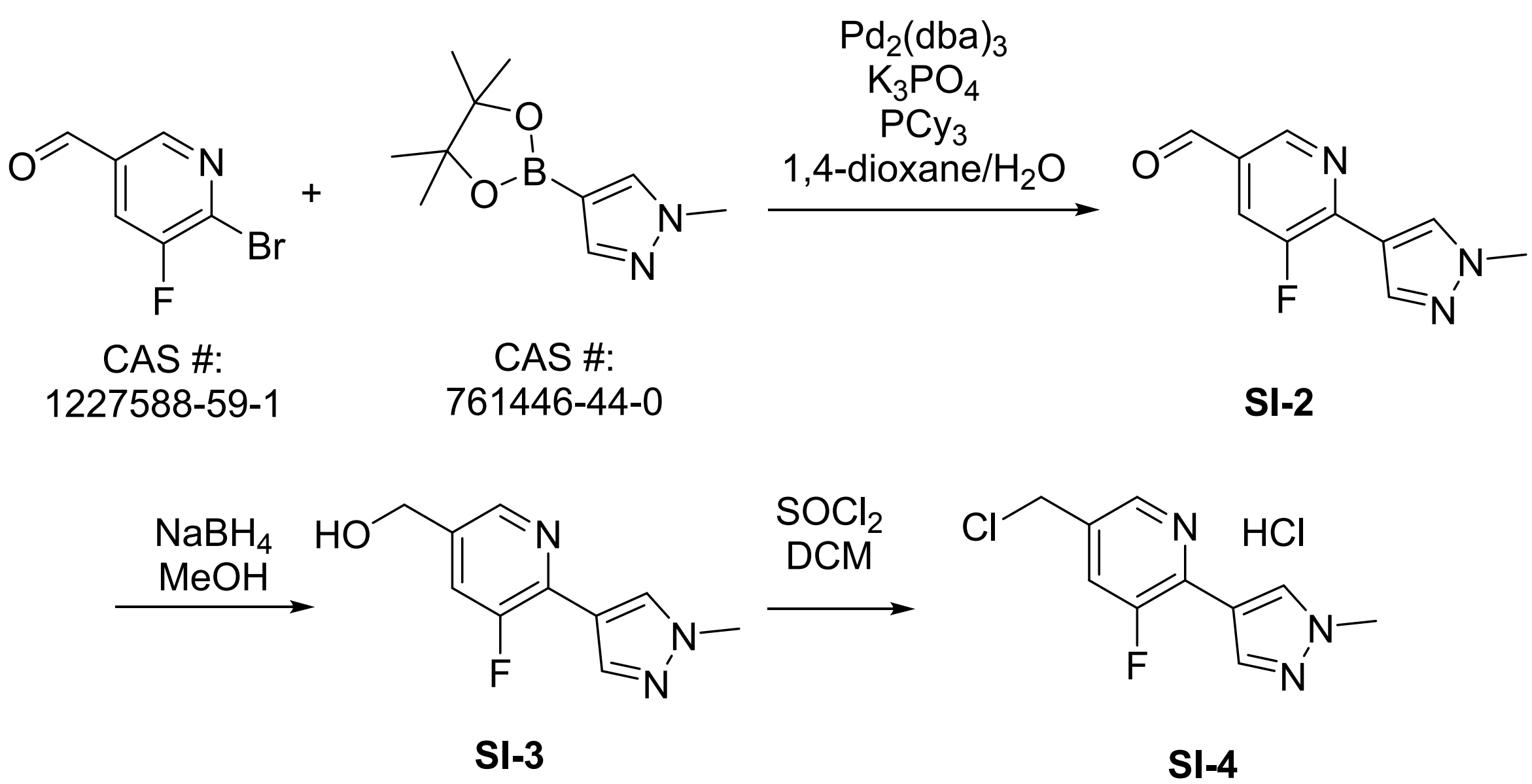

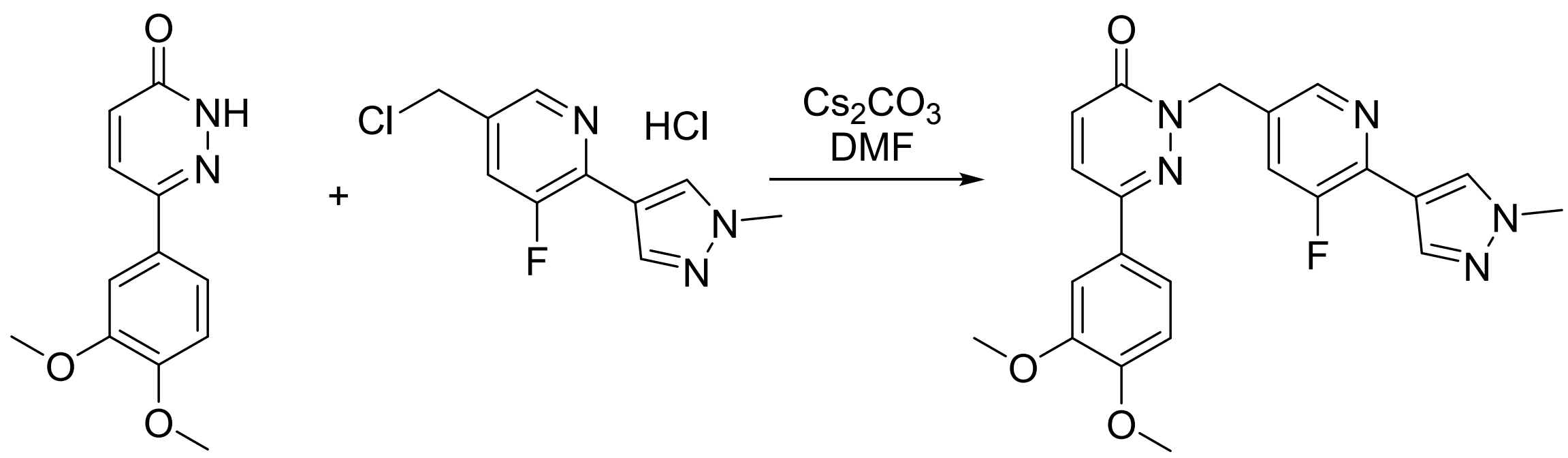

6-(3,4-Dimethoxyphenyl)pyridazin-3(2*H*)-one (SI-1). A solution of 6-bromopyridazin-3(2*H*)-one (500 mg, 2.9 mmol), (3,4-dimethoxyphenyl)boronic acid (624 mg, 3.4 mmol), Pd(dppf)Cl_2 (168 mg, 0.23 mmol), and cesium carbonate (1874 mg, 5.7 mmol) were combined in 1,4-dioxane (10 mL) and water (1 mL). The reaction was purged with nitrogen and heated with microwave irradiation to 120 °C for 15 minutes. After cooling to rt, the reaction was filtered through Celite, washing with EtOAc. Purification by column chromatography on silica gel (0-6% MeOH in DCM) afforded the title compound (582 mg, 88% yield). ^{1}H NMR (400 MHz, DMSO) δ 8.04 (d, J = 9.9 Hz, 1H), 7.45 – 7.38 (m, 2H), 7.04 (d, J = 9.0 Hz, 1H), 6.96 (dd, J = 9.9, 2.2 Hz, 1H), 3.80 (s, 6H); ES-MS $[M+H]^+$ = 233.1.

5-Fluoro-6-(1-methyl-1*H*-pyrazol-4-yl)nicotinaldehyde (SI-2). 6-Bromo-5-fluoronicotinaldehyde (530 mg, 2.6 mmol), 1-methyl-4-(4,4,5,5-tetramethyl-1,3,2-dioxaborolan-2-yl)-1*H*-pyrazole (811 mg, 3.9 mmol), $Pd_2(dba)_3$ (1510 mg, 0.13 mmol), triclycohexylphosphine (109 mg, 0.39 mmol), and potassium phosphate tribasic (1679 mg, 7.8 mmol) were combine in 1,4-dioxane (10 mL) and water (2.5 mL). The reaction was purged with nitrogen and heated with microwave irradiation to 120 ℃ for 1 hour. The reaction was cooled to ambient temperature, diluted with DCM/EtOAc, filtered over Celite and concentrated *in vacuo*. Crude residue was purified via column chromatography on silica gel (0-10% MeOH in DCM) to afford the title compound (217 mg, 97% yield). ^{1}H NMR (400 MHz, $CDCl_3$) δ 10.07 (d, J = 2.4 Hz, 1H), 8.81 (t, J = 1.7 Hz, 1H), 8.25 – 8.20 (m, 1H), 8.12 (d, J = 2.0 Hz, 1H), 7.85 (dd, J = 10.6, 1.7 Hz, 1H), 4.00 (s, 3H); ES-MS $[M+H]^+$ = 206.1.

(5-Fluoro-6-(1-methyl-1*H*-pyrazol-4-yl)pyridin-3-yl)methanol (SI-3). Sodium borohydride (286 mg, 7.6 mmol) was added to a 0 ℃ solution of 5-fluoro-6-(1-methyl-1*H*-pyrazol-4-yl)nicotinaldehyde (517 mg, 2.5 mmol) in methanol (15 mL) and the reaction stirred for 30 minutes. Acetone (1.6 mL) was added and the reaction was concentrated *in vacuo*. Purification via column chromatography on silica gel (0-10% MeOH in DCM) afforded the title compound (433 mg, 83% yield). ^{1}H NMR (400 MHz, $CDCl_3$) δ 8.34 (dq, J = 1.8, 0.9 Hz, 1H), 8.13 (dd, J = 1.8, 0.7 Hz, 1H), 8.00 (d, J = 1.9 Hz, 1H), 7.52 – 7.44 (m, 1H), 4.75 (d, J = 5.6 Hz, 2H), 3.97 (s, 3H), 1.99 (t, J = 5.8 Hz, 1H); ES-MS $[M+H]^+$ = 208.1.

5-(Chloromethyl)-3-fluoro-2-(1-methyl-1*H*-pyrazol-4-yl)pyridine; hydrogen chloride (SI-4). Thionyl chloride (0.36 mL, 5 mmol) was added dropwise to a 0 ℃ solution of (5-fluoro-6-(1-methyl-1*H*-pyrazol-4-yl)pyridin-3-yl)methanol (207 mg, 1.0 mmol) in DCM (8 mL). After 2 hours, the reaction was concentrated *in vacuo* to afford the title compound which was used without further purification (262 mg, quantitative yield). ^{1}H NMR (400 MHz, DMSO) δ 8.48 (t, J = 1.8 Hz, 1H), 8.31 (d, J = 2.2 Hz, 1H), 8.00 (dd, J = 1.7, 0.7 Hz, 1H), 7.86 (dd, J = 11.8, 1.8 Hz, 1H), 4.84 (s, 2H), 3.92 (s, 3H); ES-MS $[M+H]^+$ = 226.0.

NMR Spectra of VU6081679 (2)

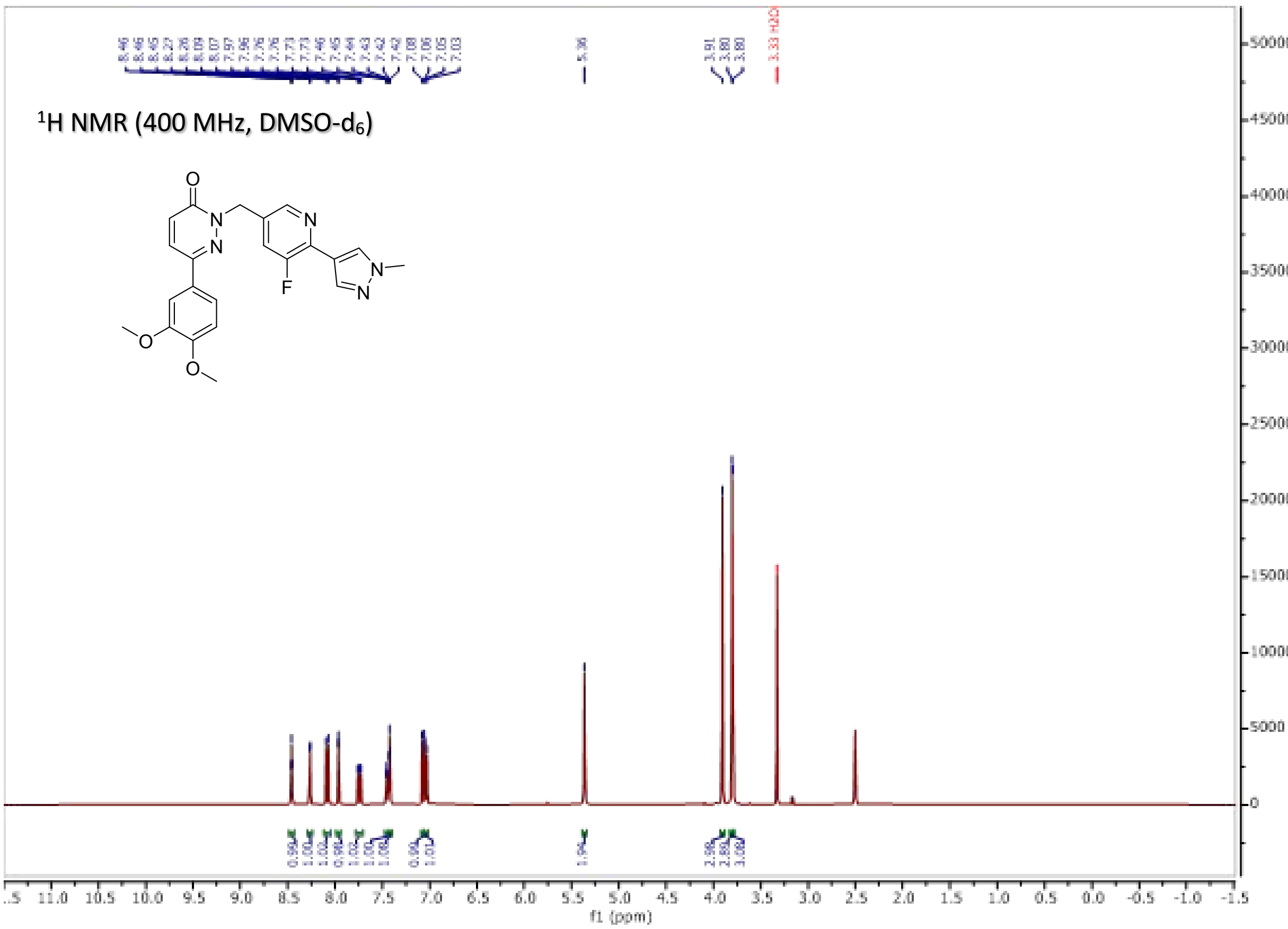

^{1}H NMR (400 MHz, DMSO-*d$_6$*) δ 8.46 (t, *J* = 1.8 Hz, 1H), 8.27 (d, *J* = 2.2 Hz, 1H), 8.09 (d, *J* = 9.8 Hz, 1H), 7.96 (dd, *J* = 1.6, 0.7 Hz, 1H), 7.75 (dd, *J* = 11.8, 1.8 Hz, 1H), 7.45 (dd, *J* = 8.4, 2.1 Hz, 1H), 7.42 (d, *J* = 2.1 Hz, 1H), 7.07 (d, *J* = 9.8 Hz, 1H), 7.05 (d, *J* = 8.5 Hz, 1H), 5.37 (s, 2H), 3.91 (s, 3H), 3.81 (s, 3H), 3.80 (s, 3H).

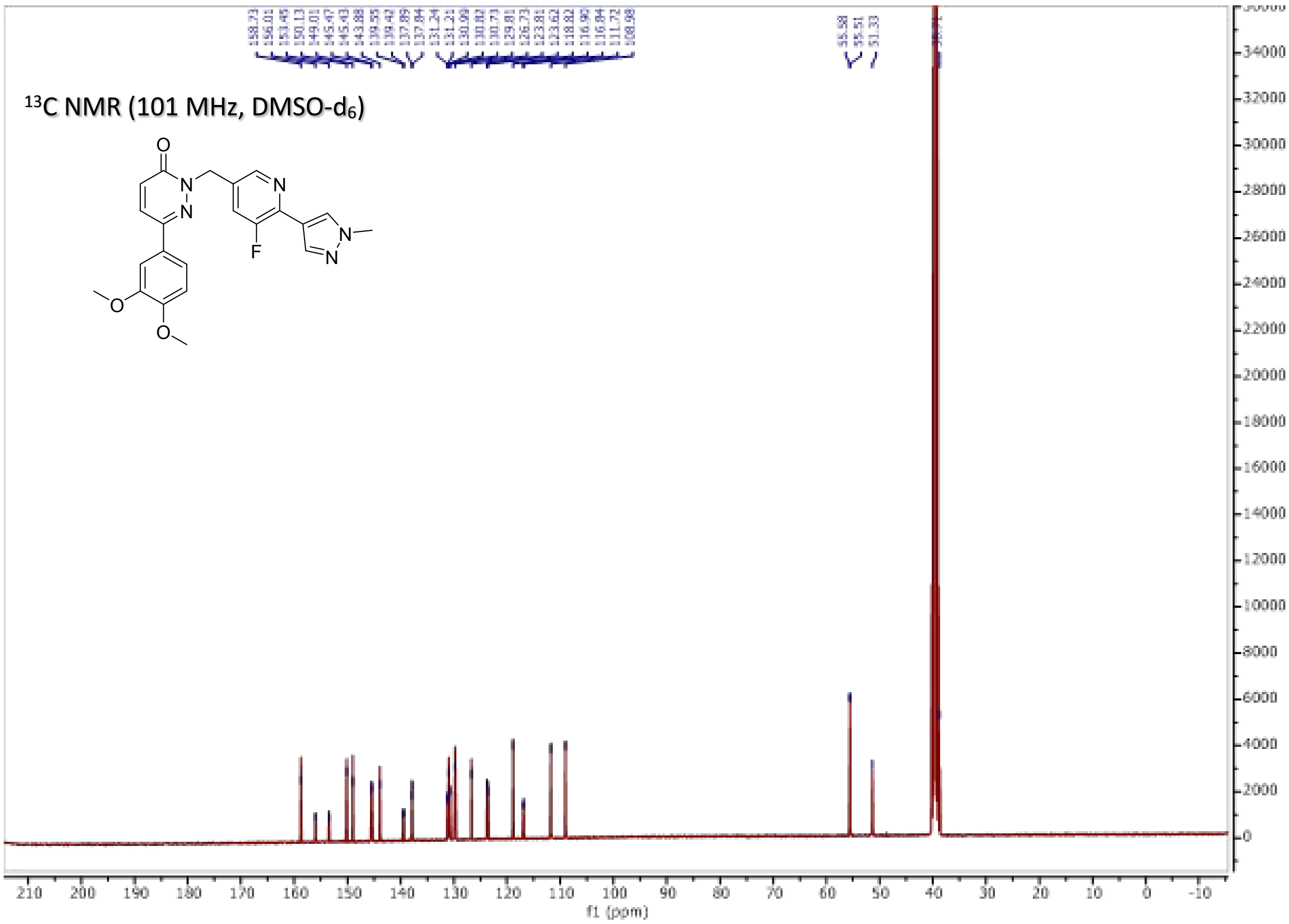

^{13}C NMR (101 MHz, DMSO-d_6) δ 158.7, 156.1, 153.5, 150.1, 149.0, 145.5, 145.4, 143.9, 139.6, 139.4, 137.9, 137.8, 131.2, 131.2, 131.0, 130.8, 130.7, 129.8, 126.7, 123.8, 123.6, 118.8, 116.9, 116.8, 111.7, 109.0, 55.6, 55.5, 51.3, 38.7.

Eurofins Lead Profiling Screen

Supplemental Table S1. Eurofins Lead Profiling Screen Data-Study FR095-0054028[1]

This is a radioligand binding panel of 44 targets including GPCRs, ion channels, transporters and nuclear hormones. Biochemical assay results are presented as the percent inhibition of specific binding at a 10 µM concentration of VU6081679 (**2**).

Target/Protein	Species	% Inhibition at 10 µM
Acetylcholinesterase	Human	3

Adenosine A_{2A}	Human	45
Adrenergic α_{1A}	Human	4
Adrenergic α_{2A}	Human	-6
Adrenergic β_1	Human	-4
Adrenergic β_2	Human	9
Androgen (Testosterone)	Human	-3
Calcium Channel L-Type, Nitrendipine	Rat	-2
Cannabinoid CB_1	Human	4
Cannabinoid CB_2	Human	4
Cholecystokinin CCK1	Human	38
Cyclooxygenase COX-1	Human	26
Cyclooxygenase COX-2	Human	14
Dopamine D_1	Human	-4
Dopamine D_{2S}	Human	-2
Endothelin ET_A	Human	-5
$GABA_A$, Flunitrazepam, Central	Rat	-1
Glucocorticoid	Human	1
Glutamate, NMDA, Glycine	Rat	-4
Histamine H_1	Human	-9
Histamine H_2	Human	-11
Lck TK Kinase	Human	-8
Monoamine Oxidase A	Rat	92
Muscarinic M_1	Human	11
Muscarinic M_2	Human	-16
Muscarinic M_3	Human	2
Nicotinic Acetylcholine $\alpha 4\beta 2$	Human	13
Opiate δ_1 (OP1, DOP)	Human	8
Opiate κ (OP2, KOP)	Human	4
Opiate μ (OP3, MOP)	Human	5
Phosphodiesterase (PDE3A)	Human	53
Phosphodiesterase (PDE4D2)	Human	101
Potassium Channel [K_{ATP}]	Rat	-3
Potassium Channel hERG	Human	8
Serotonin (5-HT_{1A})	Human	-12
Serotonin (5-HT_{1B})	Human	-4
Serotonin (5-HT_{2A})	Human	-3
Serotonin (5-HT_{2B})	Human	73
Serotonin (5-HT_3)	Human	-10
Sodium Channel, Site 2	Rat	18
Transporter, Dopamine (DAT)	Human	13
Transporter, Norepinephrine (NET)	Human	5
Transporter, Serotonin (SERT)	Human	3
Vasopressin/Oxytocin (V_{1a})	Human	3

Molecular Modeling

Structure preparation. Three cryo-EM structures of human mGlu8 were used to evaluate potential binding modes of VU6081679: antagonist-bound mGlu8 with LY341495 (PDB ID: 9MBB), agonist + PAM-bound mGlu8 with DCPG and AZ12216052 (PDB ID: 9MBC), and agonist + PAM-bound, Gi1-coupled mGlu8 with DCPG and VU0422288 (PDB ID: 9MB9).[2] Missing loop regions were modeled with MODELLER[3] using the full-length UniProt sequence as a reference. Models were inspected manually, and disulfide bond assignments were verified and corrected with pdb4amber to ensure appropriate CYX assignments before molecular dynamics simulations.

VU6081679 was prepared from an SDF file by adding explicit hydrogen atoms, removing salts and other non-defining molecular entities, neutralizing the molecule where appropriate, and generating a single three-dimensional conformer with CORINA.[4] Initial ligand placements were generated by aligning VU6081679 to the co-crystallized ligands in each mGlu8 structure, including LY341495, AZ12216052, and VU0422288. Three-dimensional ligand alignments were performed with the BioChemical Library (BCL) using the PsiFlexField method.[5] Five independent alignments were generated for each receptor state.

RosettaLigand docking. Protein-ligand docking was performed with RosettaLigand[6] using the ligand-optimized Rosetta energy function.[7] For each initial alignment, global and local conformational ensembles were generated with the BCL molecule:ConformerGenerator. Global conformers sampled ligand torsions and ring conformations broadly, whereas local conformers preserved the initial alignment geometry while refining local minima. Redundant conformers were removed by symmetry-corrected RMSD clustering using a 0.25 Å threshold.[8]

For each receptor state and conformational ensemble, 5,000 independent RosettaLigand docking trajectories were performed. Docked poses were ranked by Rosetta interface energy (interface_delta_X). Approximately 25,000 models were analyzed per receptor state. Docking convergence was assessed by plotting ligand RMSD against interface energy to identify low-energy funnels and recurrent low-RMSD pose clusters. Docking into the antagonist-bound structure served as a negative-control comparison to assess whether favorable binding was enriched in PAM-compatible receptor conformations. The top-ranked pose in the Gi-coupled

mGlu8 PAM site placed the para-hydroxyl group of VU6081679 in position to form a direct hydrogen bond with the backbone carbonyl oxygen of P582.

Ligand parameterization. Top-ranked docked poses were advanced to molecular dynamics simulations. VU6081679 was parameterized with AmberTools/AMBER24.[9,10] Ligand bond orders and formal charges were verified from the SDF file. AM1-BCC partial charges were assigned with Antechamber using the GAFF2 force field, and ligand topology and parameter files were generated with antechamber and parmchk2.[11,12]

Molecular dynamics simulations. Molecular dynamics simulations were performed with AMBER24.[13] Protein, lipid, water, and ligand interactions were described with ff14SB,[14] Lipid21,[15] TIP3P,[16] and GAFF2, respectively. Protein-ligand complexes were embedded in explicit POPC membranes and solvated with TIP3P water using Packmol-Memgen.[17] Systems were neutralized and adjusted to 150 mM NaCl.[18] Periodic boundary conditions were used with Particle Mesh Ewald treatment of long-range electrostatics and a 12 Å real-space cutoff. Covalent bonds involving hydrogen atoms were constrained with SHAKE.[19]

Each system was minimized sequentially by relaxing solvent, solute, and then the full system, with each stage consisting of 1,000 steps of steepest descent followed by 4,000 steps of conjugate-gradient minimization. Systems were heated to 100 K in the NVT ensemble for 1 ns and then to 310 K in the NPT ensemble over 1 ns using a Monte Carlo barostat with semi-isotropic pressure coupling. Hydrogen mass repartitioning was applied to non-solvent atoms to permit a 4 fs production timestep. A Langevin thermostat with a collision frequency of 1 ps^{-1} was used during heating, equilibration, and production. Five independent 1 µs production simulations were performed to assess ligand stability, pose populations, and persistence of direct and water-mediated hydrogen-bonding interactions within the mGlu8 PAM pocket.

mGlu Selectivity Assays

The mGlu subtype selectivity of compounds was determined using calcium mobilization or thallium flux using stable cell lines expressing the respective rat mGlu receptors. Calcium mobilization assays were performed for rat mGlu subtypes 1, 5, and 7. Thallium flux assays were

performed for rat mGlu subtypes 2, 3, and 4. Methods are described in detail in Henderson et al. 2026.[20]

DMPK Methods

In vitro DMPK

Hepatic microsomal intrinsic clearance

Human, rat, and mouse hepatic microsomes (0.5 mg/mL) and 1 μM test compound were incubated in 100 mM potassium phosphate pH 7.4 buffer with 3 mM $MgCl_2$ at 37 °C with constant shaking. After a 5 min preincubation, the reaction was initiated by addition of NADPH (1 mM). At selected time intervals (0, 3, 7, 15, 25, and 45 min), aliquots were taken and subsequently placed into a 96-well plate containing cold acetonitrile with internal standard (50 nM carbamazepine). Plates were then centrifuged at 3000 RCF (4° C) for 10 min, and the supernatant was transferred to a separate 96-well plate and diluted 1:1 with water for LC/MS/MS analysis. The *in vitro* half-life ($t_{1/2}$, min), intrinsic clearance (CL_{int}, mL/min/kg) and subsequent predicted hepatic clearance (CL_{hep}, mL/min/kg) were determined using Equations 1 – 3:

$$T_{1/2} = \frac{Ln(2)}{k} \qquad [1]$$

Equation 1. Determination of half-life. *k* represents the slope from linear regression analysis of the natural log percent remaining of test compound as a function of incubation time.

$$CL_{int} = \frac{0.693}{in\ vitro\ T_{1/2}}\ x\ \frac{1\ mL\ incubation}{0.5\ mg\ microsomes}\ x\ \frac{45\ mg\ microsomes}{1\ gram\ liver}\ x\ \frac{x^{a}\ gram\ liver}{kg\ body\ wt} \quad [2]$$

Equation 2. Determination of intrinsic clearance. [a]scale-up factors (gm liver / kg body weight) of 20 (human), 45 (rat), and 87.5 (mouse) were used in this calculation (Scaling factors were derived from Lin et. al.).[21]

$$CL_{hep} = \frac{Q_h * CL_{int}}{Q_h + CL_{int}} \qquad [3]$$

Equation 3. Determination of predicted hepatic clearance. Q_h represents hepatic blood flow (ml/min/kg): 21 for human, 70 for rat, and 90 for mouse.

Plasma and brain homogenate protein binding

The protein binding of each compound was determined in plasma via equilibrium dialysis employing HTDialysis membrane plates. Plasma was added to the 96 well plate containing test compound and mixed thoroughly for a final concentration of 5 μM. Subsequently, an aliquot of the plasma-compound mixture was transferred to the *cis* chamber (red) of the RED plate, with a phosphate buffer (25 mM, pH 7.4) in the *trans* chamber. The RED plate was sealed and incubated for 6 hours at 37°C with shaking (120 rpm). At completion, aliquots from each chamber transferred to a new 96 well plate and were diluted 1:1 with either plasma (*trans*) or buffer (*cis*) , at which time ice-cold acetonitrile containing internal standard (50 nM carbamazepine) (3 volumes) was added to extract the matrices. The plate was centrifuged (3000 RCF, 10 min) and supernatants transferred and diluted 1:1 (supernatant: water) into a new 96 well plate, which was then sealed in preparation for LC/MS/MS analysis. Each compound was assayed in triplicate within the same 96-well plate.

A similar approach was used to determine the degree of brain homogenate binding, which employed the same methodology and procedure with the following modifications: 1) a final compound concentration of 1 μM was used, 2) naïve rat brains were homogenized in DPBS (1:3 composition of brain: DPBS, w/w) using a Mini-Bead Beater™ machine in order to obtain blank brain homogenate, which was then treated in the same manner as the plasma samples in the previously described plasma protein binding assay.

Fraction unbound for both plasma and brain samples was determined using Equation 4.

$$f_u = \frac{Conc_{buffer}}{Conc_{plasma}} \qquad [4]$$

Equation 4 Determination of fraction unbound in plasma. The diluted fraction unbound (f_u2) in brain was calculated in the same manner by using brain homogenate rather than plasma. Undiluted fraction unbound for the brain was calculated using Equation 5

[Equation] $$f_u = \frac{1/4}{\left\{\left(\frac{1}{fu2}\right)-1\right\}+1/4} \qquad [5]$$

Equation 5 Determination of fraction unbound in brain. F_u2 represents the diluted fraction unbound.

Permeability using MDR1-MDCK cells

Cell Culture. MDCKII-MDR1 cells were cultured in media consisting of Dulbecco's Modified Eagle's Media (low glucose), 25 mM HEPES, 10% fetal bovine serum, 1% non-essential amino acids, 100 units/ml penicillin/streptomycin and 4mM G418 at 37°C, 5% CO_2 and 85% relative humidity. On day 1, MDCKII-MDR1 cells were seeded at a density of 45,000 cells/well onto Corning (Corning, NY) 24-well transwell plate (0.4 µm pore size, 0.33 cm^2 growth area) and place in the cell culture incubators. The assay was performed on day 5. The transwell plates received fresh media change one day before the experiments to prevent cell starvation.

P-glycoprotein Transwell Assay. All transwell assays were performed in HBSS buffer. Transwell assays were performed at 5 µM concentration of compounds. Transporter studies were initiated by adding dosing solutions into donor compartments and measuring appearance of compounds in receiver compartments after 120 min. Before incubation, donor samples at 0 min were collected. After 120 min of incubation, samples were collected for both donor and receiver chambers. Post-experimental incubation of lucifer yellow (100 µM) was carried out to confirm the integrity of the cell monolayer during incubation of test compounds. Quinidine and propranolol were used as P-gp substrate and high-passive permeability control respectively. All the data for controls were withing the acceptable range. The % recovery for the test compounds was above 80%.

Data Analysis. To determine apparent permeability (P_{app}) the following equation was used:

$$P_{app} = \frac{dQ}{dt} \times \frac{1}{(A \times C_0)}$$

Where dQ/dt is the rate of appearance of the test compounds in the receiver compartment, A is surface area of the membrane (0.33 cm^2), C_0 is the initial concentration (0 min) of the test compounds in the donor compartment.

The ER was calculated by the following equation:

$$Efflux\ Ratio = \frac{P_{app,\,B-A}}{P_{app,\,A-B}}$$

Where $P_{app,\ B\text{-}A}$ or $P_{app,\ A\text{-}B}$ refers to the permeability in direction of basolateral to apical (B-to-A) apical to basolateral (A-to-B), respectively.

Inhibition of Cytochrome P450 Enzymes

A cocktail of substrates for cytochrome P450 enzymes (1A2: Phenacetin, 10 µM; 2C9: Diclofenac, 5 µM; 2D6: Dextromethorphan, 5 µM; 3A4: Midazolam, 2 µM) were mixed for cocktail analysis. For P450 2C19, the substrate stock (Mephenytoin, 40 µM)and substrate mix were prepared separately for discrete analysis. The positive control for pan-P450 inhibition (miconazole) was included alongside each test compound in analysis.

A reaction mixture of 100 mM Kpi, pH 7.4, 0.1 mg/mL human liver microsomes (HLM) and Substrate Mix is prepared and aliquoted into a 96-deepwell block. Test compound and positive control (in duplicate) were then added such that the final concentration of test compound ranged from 0.1 – 30 µM. The plate was vortexed briefly and then pre-incubated at 37°C while shaking for 15 minutes. The reaction was initiated with the addition of NADPH (1 mM final concentration). The incubation continued for 8 min and the reaction quenched by 2x volume of cold acetonitrile containing internal standard (50 nM carbamazepine). The plate was centrifuged for 10 minutes (4000 rcf, 4°C) and the resulting supernatant diluted 1:1 with water for LC/MS/MS analysis. A 12 point standard curve of substrate metabolites over the range of 0.98 nM to 2000 nM.

Samples were analyzed via electrospray ionization (ESI) on an AB Sciex API-4000 (Foster City, CA) triple-quadrupole instrument that was coupled with Shimadzu LC-10AD pumps (Columbia, MD) and a Leap Technologies CTC PAL auto-sampler (Carrboro, NC). Analytes were separated by gradient elution using a Fortis C18 3.0 x 50 mm, 3 µm column (Fortis Technologies Ltd, Cheshire, UK) thermostated at 40°C. HPLC mobile phase A was 0.1% formic acid in water (pH unadjusted), mobile phase B was 0.1% formic acid in acetonitrile (pH unadjusted). The gradient started at 10% B after a 0.2 min hold and was linearly increased to 90% B over 1.2 min; held at 90% B for 0.1 min and returned to 10% B in 0.1 min followed by a re-equilibration (0.9 min). The

total run time was 2.5 min and the HPLC flow rate was 0.5 mL/min. The source temperature was set at 500°C and mass spectral analyses were performed using multiple reaction monitoring (MRM), with transitions specific for each compound utilizing a Turbo-Ionspray® source in positive ionization mode (5.0 kV spray voltage).

The IC_{50} values for each compound were obtained for the individual CYP enzymes by quantitating the inhibition of metabolite formation for each probe substrate. A 0 µM compound condition (or control) was set to 100% enzymatic activity and the effect of increasing test compound concentrations on enzymatic activity could then be calculated from the % of control activity. Curves were fitted using XLfit 5.2.2 (four-parameter logistic model, equation 201) to determine the concentration that produces half-maximal inhibition (IC_{50}).

In vivo PK

Rat IV and PO PK studies

Male Sprague-Dawley rats were used for discrete pharmacokinetic studies. Compounds in ethanol: PEG400: saline (1:4:5 v/v/v) were administered intra-venously in two rats at 1 mg/kg (IV PK). Two rats were gavaged at 10 mg/kg with compounds in 10% Tween 80 in water (PO PK). Blood samples were collected from all mice at 8 different time points post-dose (0.25, 0.50, 1.0, 2.0, 4.0, 7.0 and 24 hr) and plasma was prepared as described above. The concentration of compounds in plasma was quantified by LC/MS/MS as described below.

The IV PK experiment was used to determine clearance, Volume of distribution and half-life. The PO experiments were used to determine exposure and oral bioavailability. PK parameters were calculated using Phoenix WinNonlin software (version 8.1).

IV PK cassette and plasma-brain level determination (PBL)

In-life phase: 4 compounds plus one control were formulated as a solution in ethanol, PEG400, and DMSO (1:3:6 v/v, respectively) at a concentration of 1 mg/mL and administered as a single IV dose (1 mL/kg, 0.2 mg/kg per compound, total 1 mg/kg) to male, Sprague Dawley rats (n = 1) via injection into a surgically-implanted jugular vein catheter. Blood samples were collected

serially from a surgically implanted carotid artery catheter in each animal over multiple post-administration time points (0.033, 0.117, 0.25, 0.5, 1, 2, 4, 7, and 24 hours) into chilled, K_2EDTA anticoagulant-fortified tubes and immediately placed on wet ice. The blood samples were then centrifuged (1700 rcf, 5 minutes, 4 ˚C) to obtain plasma samples, which were stored at -80 ˚C until analysis by LC-MS/MS.

For determination of the brain over plasma ratio (Kp), the same cassette of compounds plus control were formulated in ethanol, PEG400, and DMSO (1:3:6 v/v, respectively) and administered as a single IV dose (1 mL/kg, 0.2 mg/kg per compound, total 1 mg/kg) to male, Sprague Dawley rats (n = 1) via injection into a surgically-implanted jugular vein catheter. At 15 min post dosing, blood sample was collected terminally into chilled, K_2EDTA anticoagulant-fortified tube and immediately placed on wet ice. The blood sample was then centrifuged (1700 rcf, 5 minutes, 4 ˚C) to obtain plasma sample. At the same post-administration time point, whole brain sample was obtained by rapid dissection, rinsed with PBS, and immediately frozen in individual tissue collection box (dry ice). All brain and plasma samples were stored at -80 ˚C until analysis by LC-MS/MS.

Sample Analysis by LC-MS/MS

Samples preparation for bioanalysis: Plasma samples from the in-life phase of the study were thawed at ambient temperature (benchtop), and then aliquots (20 µL per sample) were transferred to a 96-shallow-well (V-bottom) plate. Matrix-matched quality control (QC) samples and a standard curve of the compounds (1 mg/mL DMSO stock solution) were prepared in blank rat plasma (K_2EDTA-treated) or blank brain homogenate via serial dilution and transferred (20 µL each) to the plate along with multiple blank plasma and brain homogenate samples. Acetonitrile (120 µL) containing IS (10 nM carbamazepine) was added to each well of the plate to precipitate protein. The plate was then centrifuged (4000 rcf, 5 minutes, ambient temperature), and resulting supernatants (60 µL each) were transferred to a new 96-shallow-well (V-bottom) plate containing an equal volume (60 µL per well) of water (Milli-Q purified). The plate was then sealed in preparation for LC-MS/MS analysis.

Preparation of brain samples was identical to that of plasma samples except for the following modifications. While thawing, brains were weighed (inside their collection boxes using a universal empty collection box tare weight) and then subjected to mechanical homogenization (Mini-BeadBeater™, BioSpec Products, Inc., Bartlesville, OK) in the presence of zirconia/silica beads (1.0 mm) and extraction buffer (isopropanol:water, 7:3, v/v; 3 mL per sample, corrected for post-quantitation). Homogenized brain samples were then centrifuged (4000 rcf, 5 minutes, ambient temperature), and 5 uL of the supernatant was diluted in 15 uL of blank plasma for quantification of the analyte. The plasma standard curve and QCs were used for compound quantitation in brain.

LC-MS/MS analysis: Prepared samples were injected (10 µL each) onto an AB Sciex triple quad-4500 mass spectrometer system with Agilent 1290 Infinity Binary Pump and multisampler. Analytes were separated on a reverse phase column Phenomenex Kinetex® C18 (50 x 2.1 mm, 5 µm) that was thermostated at 40°C. HPLC mobile phase A was 0.5% formic acid in water (pH unadjusted); mobile phase B was 0.5% formic acid in acetonitrile (pH unadjusted). A 5% B gradient was held for 0.2 minute and was linearly increased to 90% B over 0.8 minute, with an isocratic hold for 0.5 minute, before transitioning to 10% B over 0.05 minute. The column was re-equilibrated (1 minute) before the next sample injection. The total run time was 2.55 minutes, and the HPLC flow rate was 0.5 ml/min. The source temperature was set at 500°C, and mass spectral analyses were performed using a Turbo-Ion spray source in positive ionization mode (5.0-kV spray voltage) and using multiple-reaction monitoring of transitions specific for both analytes and internal standard (m/z 237.0 to 193.9 at 25 eV). Quantitation of compounds was performed via AB Sciex Analyst software using the raw analyte:IS peak area ratios. The typical detection range was 0.5 ng/mL to ≥ 5000 ng/mL utilizing a quadratic equation regression with $1/x^2$ weighting.

Correction for dilution of all brain samples (in extraction buffer and subsequently in blank plasma, as previously described) was performed post-quantitation. The corrections for dilution in extraction buffer employed correction factors specific to brain weight and to dilution of brain extract in plasma (4 X).

Haloperidol-Induced Catalepsy in Mice

Supplemental Table 2. Time dependent effects of 2 in WT and $mGlu_8$ KO mice

	Latency to Withdraw (seconds)		
**Animal Group**	**30 minutes**	**60 minutes**	**120 minutes**
WT Veh Males (n=8)	177.0±3.0	180.0±0.0	180.0±0.0
WT 56.6 mg/kg VU6081679 Males (n=7)	50.4±9.8****	63.7±12.0****	106.0±19.5****
$mGlu_8$ KO Veh Males (n=6)	174.2±3.7	180.0±0.0	180.0±0.0
$mGlu_8$ KO VU6081679 Males (n=6)	161.8±9.2	168.5±9.3	179.7±0.33
WT Veh Females (N=3)	180.0±0.0	180.0±0.0	180.0±0.0
WT 56.6 mg/kg VU6081679 Females (n=5)	85.4±10.2****	104.0±10.7****	136.8±15.2**
$mGlu_8$ KO Veh Females (n=4)	180.0±0.0	180.0±0.0	180.0±0.0
$mGlu_8$ KO VU6081679 Females (n=4)	177.0±3.0	180.0±0.0	180.0±0.0

Supplemental Table 2. Compound **2 reverses catalepsy at 30, 60 and 120 minutes post compound dose in WT but not $mGlu_8$ KO mice**. Data were analyzed using ordinary one-way ANOVA for each sex for (**** $p < 0.0001$, $F (11, 70) = 30.53$ for males and ****$p < 0.0001$, $F (11,36) = 21.12$ for females) and then analyzed via Dunnett's post hoc test for each time point per sex. ** $p < 0.01$ and **** $p < 0.0001$. Data shown are mean +/- SEM.

References

(1) SafetyScreen44 Panel–FR. *https://www.eurofinsdiscovery.com/catalog/safetyscreen44-panel-fr/P270* (accessed 2026-05-07).

(2) Zhao, J.; Deng, Y.; Xu, Z.; Xu, C.; Zhao, C.; Li, Z.; Sun, H.; Tian, X.; Song, Y.; Cimadevila, M.; Wang, H.; Liu, Y.; Zhang, X.; Chen, Y.; Sun, S.; Yong, X.; Su, L.; He, Y.; Zhong, Y.; Yang, H.; Pin, J.-P.; Yan, W.; Shao, Z.; Liu, J. Structural Characterization of Five Functional States of Metabotropic Glutamate Receptor 8. *Mol. Cell* **2025**, *85* (18), 3460-3473.e6.

(3) Šali, A.; Blundell, T. L. Comparative Protein Modelling by Satisfaction of Spatial Restraints. *J. Mol. Biol.* **1993**, *234* (3), 779–815.

(4) Gasteiger, J.; Rudolph, C.; Sadowski, J. Automatic Generation of 3D-Atomic Coordinates for Organic Molecules. *Tetrahedron Comput. Method.* **1990**, *3* (6), 537–547.

(5) Brown, B. P.; Vu, O.; Geanes, A. R.; Kothiwale, S.; Butkiewicz, M.; Lowe, E. W.; Mueller, R.; Pape, R.; Mendenhall, J.; Meiler, J. Introduction to the BioChemical Library (BCL): An Application-Based Open-Source Toolkit for Integrated Cheminformatics and Machine Learning in Computer-Aided Drug Discovery. *Front. Pharmacol.* **2022**, *13*. 1-30.

(6) Song, Y.; DiMaio, F.; Wang, R. Y.-R.; Kim, D.; Miles, C.; Brunette, T.; Thompson, J.; Baker, D. High-Resolution Comparative Modeling with RosettaCM. *Structure* **2013**, *21* (10), 1735–1742.

(7) O'Meara, M. J.; Leaver-Fay, A.; Tyka, M. D.; Stein, A.; Houlihan, K.; DiMaio, F.; Bradley, P.; Kortemme, T.; Baker, D.; Snoeyink, J.; Kuhlman, B. Combined Covalent-Electrostatic Model of Hydrogen Bonding Improves Structure Prediction with Rosetta. *J. Chem. Theory Comput.* **2015**, *11* (2), 609–622.

(8) Mendenhall, J.; Brown, B. P.; Kothiwale, S.; Meiler, J. BCL::Conf: Improved Open-Source Knowledge-Based Conformation Sampling Using the Crystallography Open Database. *J. Chem. Inf. Model.* **2021**, *61* (1), 189–201.

(9) Case, D. A.; Cerutti, D. S.; Cruzeiro, V. W. D.; Darden, T. A.; Duke, R. E.; Ghazimirsaeed, M.; Giambaşu, G. M.; Giese, T. J.; Götz, A. W.; Harris, J. A.; Kasavajhala, K.; Lee, T.-S.; Li, Z.; Lin, C.; Liu, J.; Miao, Y.; Salomon-Ferrrer, R.; Shen, J.; Snyder, R.; Swails, J.; Walker, R. C.; Wang, J.; Wu, X.; Zeng, J.; Cheatham III, T. E.; Roe, D. R.; Roitberg, A.; Simmerling, C.; York, D. M.; Nagan, M. C.; Merz, K. M. Recent Developments in Amber Biomolecular Simulations. *J. Chem. Inf. Model.* **2025**, *65* (15), 7835–7843.

(10) Case, D. A.; Aktulga, H. M.; Belfon, K.; Cerutti, D. S.; Cisneros, G. A.; Cruzeiro, V. W. D.; Forouzesh, N.; Giese, T. J.; Götz, A. W.; Gohlke, H.; Izadi, S.; Kasavajhala, K.; Kaymak, M. C.; King, E.; Kurtzman, T.; Lee, T.-S.; Li, P.; Liu, J.; Luchko, T.; Luo, R.; Manathunga, M.; Machado, M. R.; Nguyen, H. M.; O'Hearn, K. A.; Onufriev, A. V.; Pan, F.; Pantano, S.; Qi, R.; Rahnamoun, A.; Risheh, A.; Schott-Verdugo, S.; Shajan, A.; Swails, J.; Wang, J.; Wei, H.; Wu, X.; Wu, Y.; Zhang, S.; Zhao, S.; Zhu, Q.; Cheatham, T. E.; Roe, D. R.; Roitberg, A.; Simmerling, C.; York, D. M.; Nagan, M. C.; Merz, K. M. AmberTools. *J. Chem. Inf. Model.* **2023**, *63* (20), 6183–6191.

(11) Wang, J.; Wang, W.; Kollman, P. A.; Case, D. A. Automatic Atom Type and Bond Type Perception in Molecular Mechanical Calculations. *J. Mol. Graphics Modell.* **2006**, *25* (2), 247–260.

(12) Wang, J.; Wolf, R. M.; Caldwell, J. W.; Kollman, P. A.; Case, D. A. Development and Testing of a General Amber Force Field. *J. Comput. Chem.* **2004**, *25* (9), 1157–1174.

(13) D. A. Case, K. Belfon, I. Y. Ben-Shalom, S. R. Brozell, D. S. Cerutti, T. E. Cheatham, III, V. W. D. Cruzeiro, T. A. Darden, R. E. Duke, G. Giambasu, M. K. Gilson, H. Gohlke, A. W. Goetz, R. Harris, S. Izadi, S. A. Izmailov, K. Kasavajhala, A. Kovalenko, R. Krasny, T. Kurtzman, T. S. Lee,

S. LeGrand, P. Li, C. Lin, J. Liu, T. Luchko, R. Luo, V. Man, K. M. Merz, Y. Miao, O. Mikhailovskii, G. Monard, H. Nguyen, A. Onufriev, F. Pan, S. Pantano, R. Qi, D. R. Roe, A. Roitberg, C. Sagui, S. Schott-Verdugo, J. Shen, C. L. Simmerling, N. R. Skrynnikov, J. Smith, J. Swails, R. C. Walker, J. Wang, L. Wilson, R. M. Wolf, X. Wu, Y. Xiong, Y. Xue, D. M. York, and P. A. Kollman. 2020. AMBER 2020. University of California, San Francisco.

(14) Maier, J. A.; Martinez, C.; Kasavajhala, K.; Wickstrom, L.; Hauser, K. E.; Simmerling, C. Ff14SB: Improving the Accuracy of Protein Side Chain and Backbone Parameters from Ff99SB. *J. Chem. Theory Comput.* **2015**, *11* (8), 3696–3713.

(15) Dickson, C. J.; Walker, R. C.; Gould, I. R. Lipid21: Complex Lipid Membrane Simulations with AMBER. *J. Chem. Theory Comput.* **2022**, *18* (3), 1726–1736.

(16) Jorgensen, W. L.; Chandrasekhar, J.; Madura, J. D.; Impey, R. W.; Klein, M. L. Comparison of Simple Potential Functions for Simulating Liquid Water. *J. Chem. Phys.* **1983**, *79* (2), 926–935.

(17) Schott-Verdugo, S.; Gohlke, H. PACKMOL-Memgen: A Simple-To-Use, Generalized Workflow for Membrane-Protein–Lipid-Bilayer System Building. *J. Chem. Inf. Model.* **2019**, *59* (6), 2522–2528.

(18) Joung, I. S.; Cheatham, T. E. Determination of Alkali and Halide Monovalent Ion Parameters for Use in Explicitly Solvated Biomolecular Simulations. *J. Phys. Chem. B* **2008**, *112* (30), 9020–9041.

(19) Miyamoto, S.; Kollman, P. A. Settle: An Analytical Version of the SHAKE and RATTLE Algorithm for Rigid Water Models. *J. Comput. Chem.* **1992**, *13* (8), 952–962.

(20) Henderson, S. H.; Ringuette, A. E.; Whomble, D. L.; Capstick, R. A.; Richardson, A. E.; Maurer, M. A.; Billard, N. B.; Lei, X.; Wilkinson, J. C.; Kethanapalli, S. H.; Cho, H. P.; Rodriguez, A. L.; Niswender, C. M.; Peng, W.; Rook, J. M.; Chang, S.; Blobaum, A. L.; Boutaud, O.; Felts, A. S.; Conn, P. J.; Lindsley, C. W.; Temple, K. J. Discovery of 7-(Pyridin-3-yl)Thieno[3,2-*b*]Pyridine-5-Carboxamides as Negative Allosteric Modulators of Metabotropic Glutamate Receptor Subtype 5. *ACS Chem. Neurosci.* **2026**, *17* (3), 610–623.

(21) Lin, J. H.; Chiba, M.; Balani, S. K.; Chen, I. W.; Kwei, G. Y.; Vastag, K. J.; Nishime, J. A. Species Differences in the Pharmacokinetics and Metabolism of Indinavir, a Potent Human Immunodeficiency Virus Protease Inhibitor. *Drug Metab. Disp.* **1996**, *24* (10), 1111–1120.
